# Unveiling the role of *srbA* sRNA in biofilm formation by regulating *algU*, *mucA*, *rhlA*, and *rsmA* in *Pseudomonas aeruginosa*

**DOI:** 10.1042/BCJ20240650

**Published:** 2025-05-23

**Authors:** Piyali Saha, Samir Kumar Mukherjee, Sk Tofajjen Hossain

**Affiliations:** Department of Microbiology, University of Kalyani, Kalyani 741235, West Bengal, India

**Keywords:** biofilm, gene expression, *Pseudomonas aeruginosa*, small RNA, *srbA*

## Abstract

The survival and increasing antimicrobial resistance of various bacteria, including clinically relevant opportunistic pathogen, *Pseudomonas aeruginosa*, largely depends on their biofilm architectural strength that makes a challenge to eradicate it. Small RNAs (sRNAs) have been identified as the key modulators in regulating the expression and function of different transcriptional regulators, and the components of regulatory networks involved in bacterial biofilm formation. This study was focused to identify the regulatory role of the *srbA* sRNA in controlling biofilm formation in *P. aeruginosa*. *srbA* was found to be up-regulated in both substratum-attached and colony biofilms compared with planktonic growth conditions. Further analysis revealed that *srbA* overexpressing strain produced more biofilm, whereas a significant reduction in biofilm formation was noted due to *srbA* deletion. Interestingly, it was also predicted from the study that *srbA* might regulate the expression of AlgU/MucA, the sigma and anti-sigma factor, involved in biofilm developmental network. Additionally, *srbA* showed possible interference on the expression of two other important biofilm regulatory genes, *rhlA* and *rsmA*. Overall, this research highlights the critical role of *srbA* sRNA as a central regulator of biofilm formation and possibly the pathogenicity of *P. aeruginosa*. These findings might offer potential avenues for developing targeted therapeutic strategies to mitigate biofilm-related infections caused by *P. aeruginosa*.

## Introduction

*Pseudomonas aeruginosa* is an opportunistic bacterial pathogen for causing secondary infections in hospital settings, raising significant global public health concerns as highlighted in the WHO’s 2024 bacterial priority pathogens list [1]. Its ability to form robust biofilms, to adapt swiftly, and to exhibit high levels of inherent resistance to antimicrobial has positioned it as a critical pathogen across a wide variety of natural and artificial environments, together with various indwelling medical devices [2]. Biofilm-associated infections pose a major threat to human health in the era of rising antimicrobial resistance [3,4]. Although the genetic mutations often underlie antimicrobial resistance, biofilms also offer an adaptive resistance. Even bacteria sensitive to antibiotics could escape the effect in a biofilm state, however, they may return to a susceptible form once the biofilm disperses [5,6].

Population density-dependent cell–cell communication, referred to as quorum sensing (QS), is essential for the formation of biofilms by *P. aeruginosa* [7,8]. QS system plays a pivotal in the pathogenesis of *P. aeruginosa*, starting from the initial colonization in the host to subsequent invasion, infection, spread, evasion of the immune response, and development of drug resistance [9]. The four primary and interconnected QS networks in *P. aeruginosa* include the Las, Rhl, Pqs, and Iqs systems. These systems function through a hierarchical framework, facilitating intricate interactions among various cellular signals [10]. The Las system occupies the highest position in this hierarchy, overseeing the regulation of the other QS systems, while the Rhl system is positioned beneath. The Pqs system activates the Rhl system and is under the regulation of Las, whereas Iqs governs both the Pqs and Rhl systems and is itself activated by Las [8,11]. Through this complex network, the activated Rhl system controls the production of various QS-related virulence factors [10,12]. The relationship between QS system and biofilm formation is indirectly influenced by the nature of motility, as well as the production of various components of biofilm matrix [5]. Swarming motility, characterized as a coordinated feature of surface movement, is particularly significant during the initial phase of biofilm development and is regulated by the Rhl system in *P. aeruginosa* [13,14].

A complex regulatory network, acting via transcriptional, post-transcriptional, and post-translational processes in response to environmental and host-derived signals through its QS-system, modulates *P. aeruginosa*’s adaptability and pathogenicity [5,8]. The AlgU (σ^22^) sigma factor, a stress response master regulator and functional counterpart of *Escherichia coli* σ^E^, coordinates nearly 300 regulatory genes and plays a crucial role in regulating synthesis of virulence factors and other infection-related processes by affecting QS network [15–18]. AlgU enhances alginate production by up-regulating the *algD* operon and activating transcription factors AlgR and AmrZ, those are pivotal for alginate synthesis in mucoid strains [16,17]. The transition from the nonmucoid to the mucoid phenotype is accompanied by a cascade of genetic changes resulting from *mucA* mutations [17,18]. Under stress-free conditions, AlgU is inhibited by MucA, whereas under stress AlgU becomes relieved to trigger alginate production. *mucA-*mutant strains of *P. aeruginosa* have been reported to show increased alginate synthesis with increased biofilm forming ability and stress resistance [19–21]. The Rhl system of *P. aeruginosa* regulates biofilm formation through the RhlA protein, which synthesizes rhamnolipids, a glycolipid essential for maintaining the biofilm matrix [5,8,22,23]. The autoinducer binds to RhlR, resulting enhanced expression of *rhlA*, which is crucial for the production of rhamnolipids [24]. Rhamnolipids not only support biofilm structure but also facilitate bacterial dispersion, allowing *P. aeruginosa* to occupy new ecological niche [25,26]. RsmA, another key regulator in *P. aeruginosa*, belongs to the CsrA family of RNA-binding proteins, which has been reported to control virulence, motility, biofilm formation, and metabolism by interacting with the target mRNAs [27–29].

Recently, non-coding small RNAs (sRNAs) have been reported to act as the crucial regulator for the adaptability of *P. aeruginosa*, including biofilm development and pathogenesis [30–33]. The Gac/Rsm signaling cascade promotes the production of *RsmY* and *RsmZ*, both subsequently relieve the repression by RsmA on the target genes, and thereby facilitates biofilm development [34]. Another sRNA, *PhrS*, was reported to regulate the QS system regulator PqsR (MvfR), which is essential for biofilm formation via the PQS signaling pathway [35]. The *CrcZ* sRNA is the part of the carbon catabolite repression system and affects biofilm formation by inhibiting *Crc* expression, especially when carbon sources are limited [36]. Recently, regulatory role of *PA0730*.1 sRNA on the expression of different traits of *P. aeruginosa* has also been reported to be linked with pathogenicity and biofilm formation [32]. The *srbA* sRNA was earlier reported to up-regulate during the stationary phase and biofilm formation in *P. aeruginosa* PA14, though its precise function in biofilm regulation remained unclear [37]. More recently, it has been documented that *srbA* could regulate genes encoding the major enzymes involved in the TCA cycle, thus is responsible in nutritional adaptation in *P. aeruginosa* PAO1, which was further reported to be linked with the production of various virulence factors [33].

With this background, the present study was aimed to investigate the possible role of the *srbA* sRNA in biofilm formation and its regulatory influence on various biofilm-controlling factors by studying *srbA* deletion and overexpression strains to unveil its molecular targets. Understanding the role of *srbA* could reveal new insights into the regulation of biofilm development that could help to manage biofilm-associated infections by *P. aeruginosa*.

## Results

### The expression level of *srbA* in biofilm state

*Pseudomonas* genome database reveals the existence of a 239 bp span encoding *srbA* sRNA, resulting from the transcription of the reverse strand of a locus in between *aceA* and *PA2633* genes of *P. aeruginosa* PAO1 [33,38], that was earlier coined as *pant235* [39] and *PA2633*.1 [40]. Additionally, *srbA* sequence data obtained from PAO1 were reported as conserved among other strains of *P. aeruginosa*, including that in PA14, termed as *PA14sr_067* [33,41]. Higher expression level of *srbA* was earlier documented under biofilm state of *P. aeruginosa* PA14 [37]; however, the regulatory role of *srbA* on biofilm development is still unclear.

**Figure 1:**
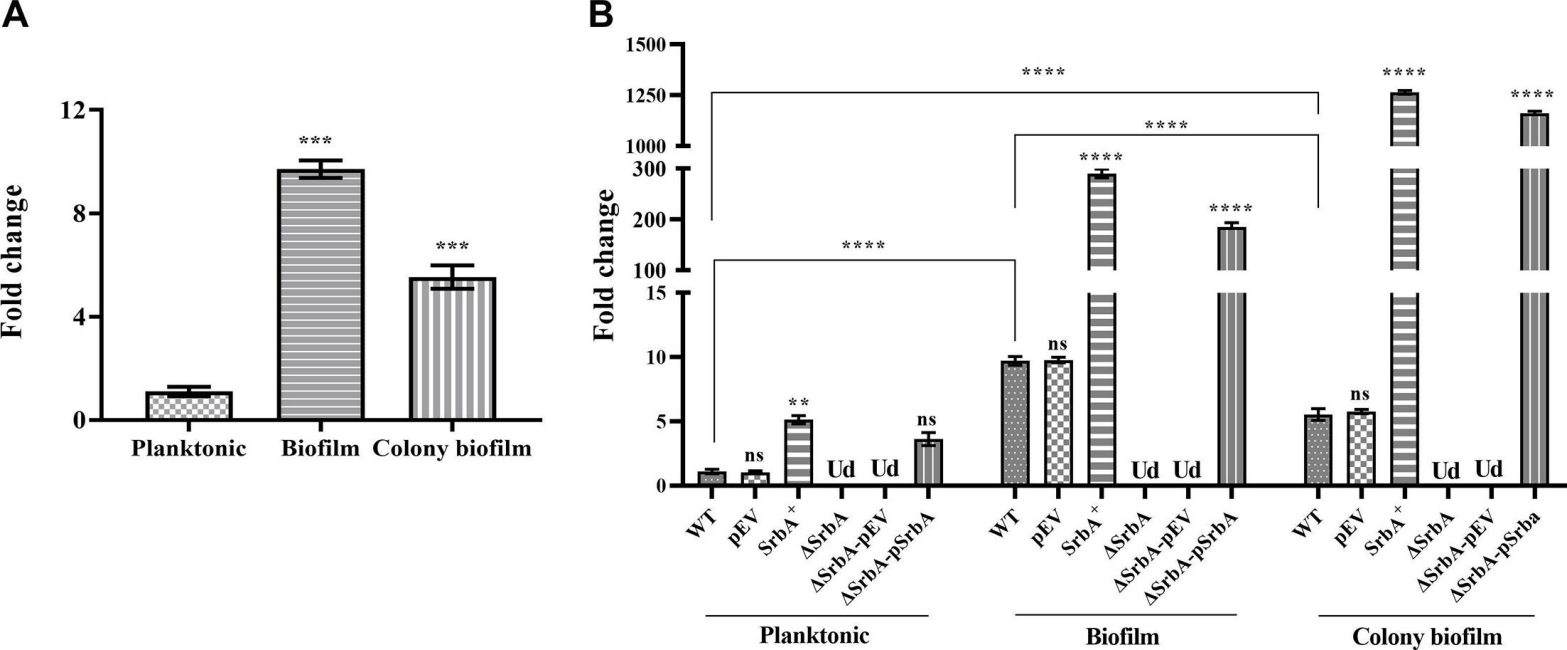
The relative abundance of *srbA* in *P. aeruginosa* under various growth conditions. (**A**) Comparative *srbA* expression among the substratum-attached biofilm, colony biofilm, and mid-log phase planktonic growth. (**B**) *srbA* expression in wildtype (WT), empty vector control (pEV), overexpression (SrbA^+^), deletion (ΔSrbA), deletion empty vector control (ΔSrbApEV), and complementation of *srbA* deletion (ΔSrbApSrbA) strains under mid-log planktonic, substratum attached biofilm, and colony biofilm conditions. Fold changes in expression were calculated relative to the WT strain grown in planktonic conditions, regardless of growth state. The level of quantitative expression of *srbA* was determined using *rpoD* as the reference house-keeping gene for the normalization. Statistical analysis was performed using one-way ANOVA for each condition, comparing the mean of each group with that of the WT strain. Additionally, the expression levels of the WT strain in different conditions were compared across all groups. Data presented are the mean of three replicates with ±SEM. ‘Ud’ and ‘ns’ stand for undetermined and non-significant, respectively. Statistical significance is indicated by **P<0.01 and ****P<0.0001.

In this study, expression levels of *srbA* sRNA in *P. aeruginosa* PAO1 were studied during substratum-attached biofilm and colony biofilm states and were compared with that in the mid-log planktonic stage. The RT-qPCR data revealed increase level of *srbA* by ~5.5-fold in colony biofilm and ~9.7-fold in substratum biofilm states in comparison with that in the planktonic cells (Figure 1A). Additionally, the abundance of *srbA* was quantitatively analyzed using *srbA* overexpression strain SrbA^+^, *srbA* deleted strain ΔSrbA, and complementation of deletion strain ΔSrbApSrbA, and their respective control strains, pEV, wildtype (WT), and ΔSrbApEV. In planktonic state, the expression of the *srbA* was found to be ~5.1 and ~3.6-fold higher in SrbA^+^ and ΔSrbApSrbA, respectively, compared with the WT strain.

In substratum-attached biofilm state, *srbA* levels were elevated by ~29.9 and ~19.1-fold in SrbA^+^ and ΔSrbApSrbA, respectively, compared with the WT strain. In contrast, abundance of *srbA* was increased by ~229.9 and ~211-fold in SrbA^+^ and ΔSrbApSrbA, respectively, in comparison with the WT strain under colony biofilm state. Within each growth condition, WT and pEV strains showed similar levels of *srbA* (Figure 1B).

### Role of *srbA* on *P. aeruginosa* biofilm formation

**Figure 2:**
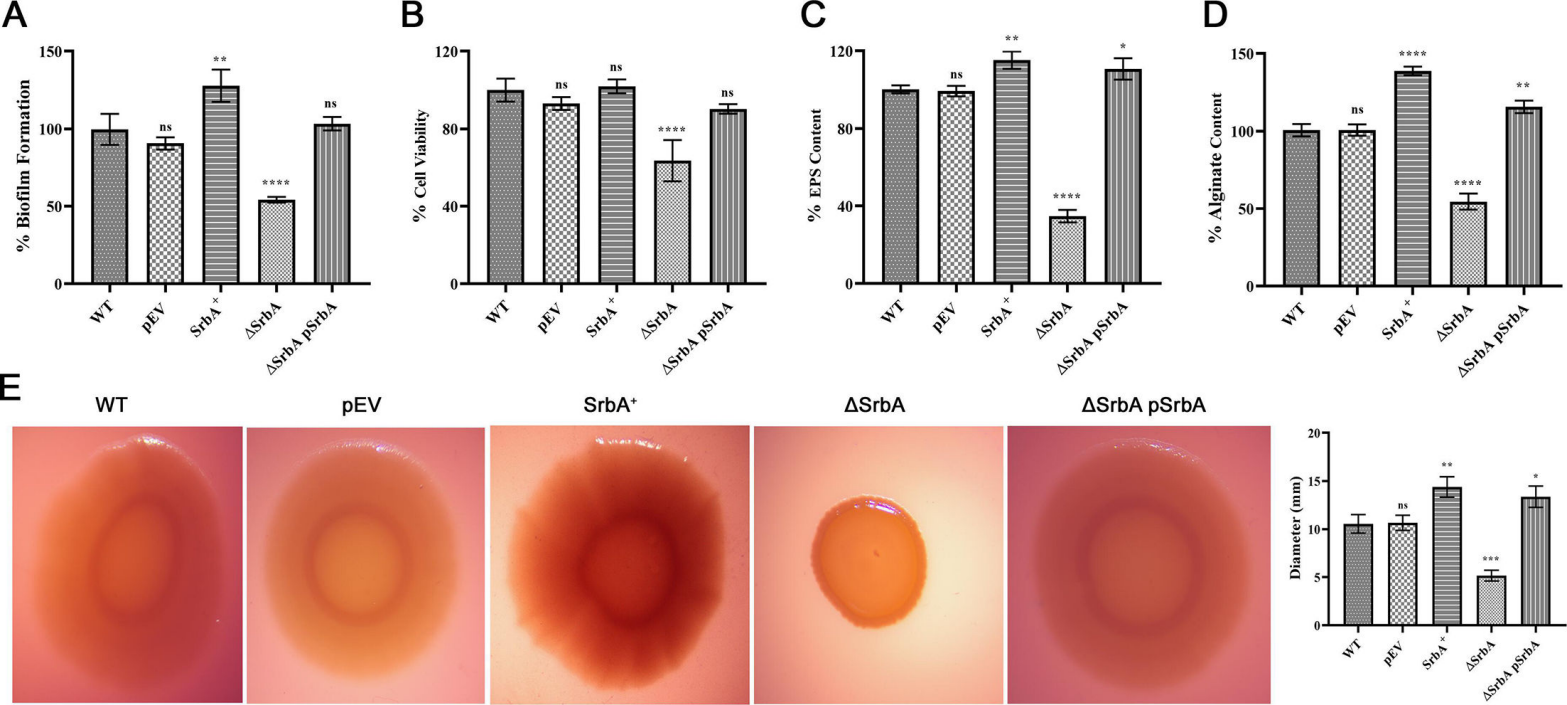
Involvement of *srbA* sRNA in biofilm formation and survival of *P. aeruginosa*. (**A**) Biofilm forming capability of WT was measured by CV assay and compared with that of the pEV, SrbA^+^, ΔSrbApSrbA, and ΔSrbA strains. (**B**) The cell viability of WT strains in the biofilm, as measured by MTT assay, was compared with the pEV, SrbA^+^, ΔSrbA, and ΔSrbApSrbA strains. (**C**) Amount of EPS in the biofilm matrix of WT strains, as determined by Congo red staining, was compared with the pEV, SrbA^+^, ΔSrbA, and ΔSrbApSrbA strains. (**D**) Amount of alginate determined by carbazole assay in the biofilm of pEV, SrbA^+^, ΔSrbA, and ΔSrbApSrbA strains in comparison with the WT strain. (**E**) Comparative morphological nature of the colonies as appeared on the Congo red agar plates of WT, pEV, SrbA^+^, ΔSrbA, and ΔSrbApSrbA strains, with a quantitative illustration of respective colony diameters. Data presented are the mean of three replications with ±SEM; statistical analysis was performed using one-way ANOVA for each condition, comparing the mean of each group with that of the WT strain. ‘ns’ indicates non-significance, and *, **, ***, and **** correspond to significance at *P*<0.1, 0.01, 0.001, and 0.0001, respectively. sRNA, small RNA; WT, wildtype.

The contribution of *srbA* on the biofilm formation in *P. aeruginosa* was analyzed using SrbA^+^, ΔSrbA, and ΔSrbApSrbA, and respective control strains pEV, WT, and ΔSrbApEV. Crystal violet assay for biofilm formation revealed that the SrbA^+^ produced significantly ~27% more biofilm compared to the WT, whereas deletion of *srbA* caused a significant decrease in biofilm formation by ~46%. Biofilm forming ability restored in ΔSrbApSrbA construct after plasmid-mediated reintroduction of *srbA* in deletion strain (Figure 2A). Viable cell mass within the biofilms, as measured by MTT assay, significantly declined (~37%) in ∆SrbA which was restored in ΔSrbApSrbA strain; however, no significant alteration in cell viability was observed due to overexpression of *srbA* compared with the WT strain (Figure 2B). The EPS content could indicate the amount of biofilm formed and was quantified using Congo red binding method. Overexpression of the *srbA* resulted ~15% increase in EPS production; on the contrary, it was sharply decreased (~65%) due to the deletion of *srbA*; however, it was found to be restored in the ΔSrbApSrbA strain, resembling that in the SrbA^+^ (Figure 2C). Biofilm maturation and its structural integrity largely depend on the amount of alginate present in the matrix; thus, the alginate was quantified in all the test strains. The SrbA^+^ strain exhibited an ~38% increase in alginate production compared with the pEV strain. In contrast, the ∆SrbA showed about a ~46% reduction in alginate levels in comparison with the WT strain. However, the complementation of deletion strain ΔSrbApSrbA was able to restore alginate production to a level comparable with those of the WT strain (Figure 2D).

Apparently, slime layer in the bacterial colonies reflects the quantitative level of EPS in the colony biofilm, which was visualized on tryptone agar plates having Congo red, that binds to EPS within the slime layer resulting characteristic colony morphology with red ring around, after an incubation period of 24 h at 37°C. Visibly, SrbA^+^ colony showed higher density of bound dye, suggesting presence of more EPS; on the contrary, ∆SrbA exhibited different colony morphology with smaller diameter and absence of red ring that might be due to the less amount EPS (Figure 2E). Effect of deletion was found to be reversed in ΔSrbApSrbA and resembled the colony morphology and diameter like the WT, and SrbA^+^ strains. Overall, WT and pEV strains showed similar features as far as their EPS and biofilm production abilities are concerned.

### Role of *srbA* on biofilm architecture of *P. aeruginosa*

**Figure 3:**
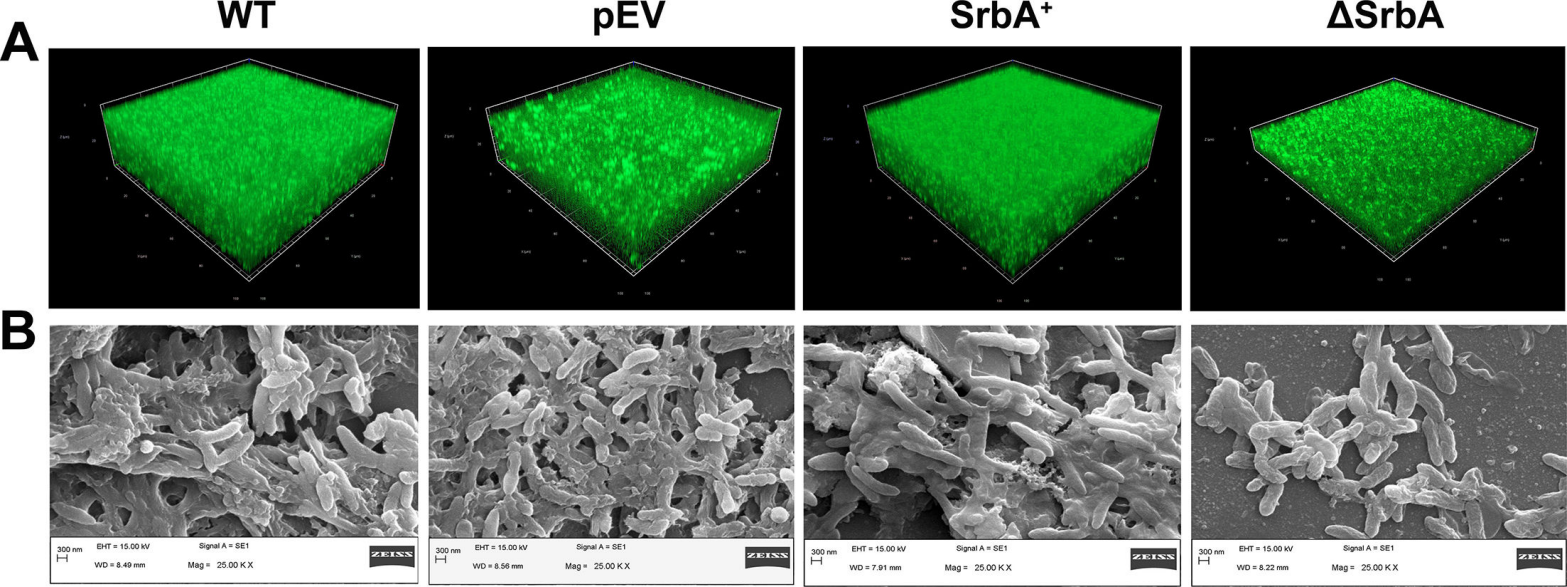
Comparative microscopic study of biofilm architecture of *P. aeruginosa* WT, pEV, SrbA^+^, and, ΔSrbA strains. (**A**) Confocal laser scanning micrographs of the strains harboring pUCP30T-eCFP, were captured for the analysis of biofilm thickness. (**B**) Scanning electron microscopic images of the strains for visualization of biofilm architecture. Data are representative of three observations. WT, wildtype.

Biofilm formation capacity and architectural feature of both of SrbA^+^ and ΔSrbA were visualized by confocal scanning laser microscopy (CSLM) and scanning electron microscopy (SEM), and comparative analysis was done with their respective control strains, pEV and WT. For CSLM study, *P. aeruginosa* harboring pUCP30T-eCFP was used to qualitative measurement of cell density in the biofilm by eCFP fluorescence. CSLM images revealed almost similar biofilm thickness in WT and pEV strains, and overexpression *srbA* resulted thicker and dense biofilm in SrbA^+^ strain with higher Z stack; on the contrary, deletion of *srbA* resulted significant decrease in bio-volume and biofilm thickness with thinner Z stack (Figure 3A and Table 1). Having consistency with CLSM study, SEM images of both WT and pEV also showed biofilm architecture with highly populated bacterial cells in the well-organized matrix. SEM image of the SrbA^+^ strain showed cells with slightly larger in length, embedded in higher amount of EPS matrix. In contrast, the ΔSrbA strain failed to develop such a complex biofilm architecture, indicating that the absence of *srbA* severely affected the formation structured biofilms (Figure 3B).

**Table 1:** COMSTAT analysis of the biofilm images captured by CLSM.

Strain name	Bio-volume (µm^3^/µm^2^)^1^	Average thickness (µm)^1^
WT	9.03976 ± 0.80076	31.95 ± 0.93
pEV	10.88244 ± 0.2998	27.63 ± 0.46
SrbA^+^	9.29118 ± 0.96684	32.49 ± 0.41
ΔSrbA	5.43842 ± 0.54422	11.43 ± 0.96

1 Data are the mean from three replications with ±SD.

### Regulatory role of *srbA* on biofilm-controlling gene expression

**Figure 4:**
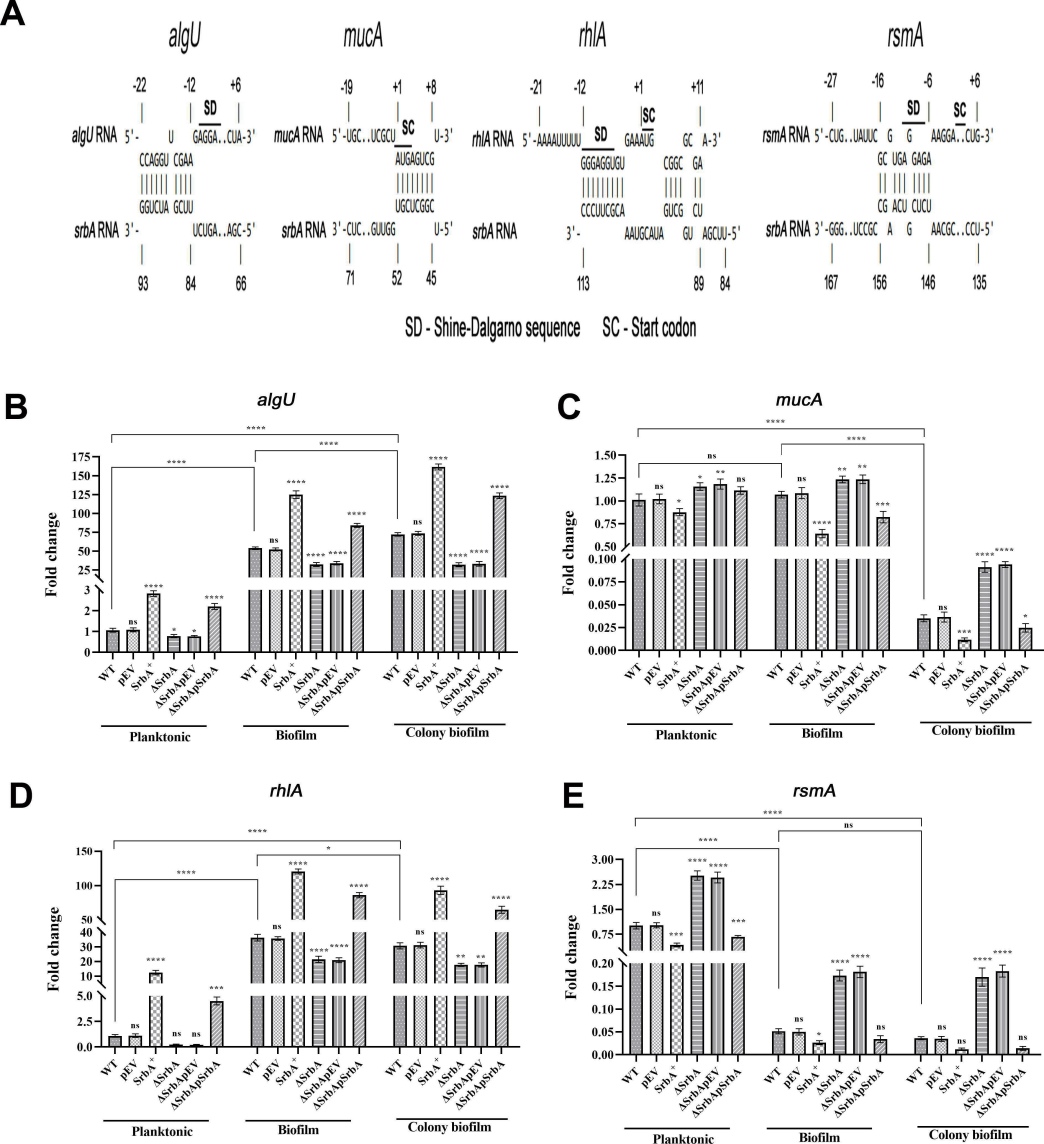
*srbA* regulates the genes associated with *P. aeruginosa* biofilm formation. (**A**) Interaction of *srbA* sRNA with the regulatory region near the start codon and Shine-Dalgarno sequence of *algU*, *mucA*, *rhlA*, and *rsmA*, as analyzed by IntaRNA software. RT-qPCR analysis of WT, pEV, SrbA^+^, ΔSrbA, ΔSrbApEV, and ΔSrbApSrbA cells grown in planktonic, substratum-attached biofilm, and colony biofilm states for the expression study of (**B**) *algU*, (**C**) *mucA*, (**D**) *rhlA*, and (**E**) *rsmA*, taking *rpoD* as a reference gene. Statistical analysis was performed by one-way ANOVA for each gene under specified condition, comparing the mean of each condition with that of the WT strain. Additionally, the expression levels of the WT strain in different conditions were compared across all groups. Data presented are the mean of three replications with ±SEM; ‘ns’ indicates non-significance, and *, **, ***, and **** correspond to significance at *P*<0.1, 0.01, 0.001, 0.0001, respectively. sRNA, small RNA; WT, wildtype.

Observing the involvement of *srbA* in the biofilm formation by *P. aeruginosa*, emphasis was given to find out the genetic target of *srbA* among different biofilm regulatory genes. Accordingly, base-pair complementarity analyses of *srbA* sRNA with mRNA region containing Shine–Dalgarno (SD) sequence and start codon of different biofilm regulatory genes, such as *algD* (-23 to +52), *algU* (-22 to +6), *fimX* (-54 to +21), *fleQ* (-57 to +18), *mucA* (-19 to +8), *pelA* (-40 to +35), *pslA* (-37 to +38), *pslB* (-54 to +21), *rhlA* (-21 to +11), and *rsmA* (-27 to +6), were done using IntaRNA 2.0 using the default settings (Supplementary Figure S1) [8,10,14,15,18,21,24,27,42]. Based on significant matching with the regulatory region near the start codon and SD-sequence, *algU*, *mucA*, *rhlA*, and *rsmA* were selected for their expressional study (Supplementary Figure S2 and S3). The sequence complementarity analysis showed that 84–93 bp, 45–52 bp, 89–113 bp, and 146–156 bp sequences of *srbA* paired with the transcript regions of *algU* (-22 to -12), *mucA* ( + 1 to + 8), *rhlA* (-12 to +11), and *rsmA* (-16 to -6), respectively, suggesting a possible function of *srbA* in regulating the expression of these genes (Figure 4A). The expression levels of *algU*, *mucA*, *rhlA*, and *rsmA* were measured in the WT, pEV, SrbA^+^, ΔSrbA, ΔSrbApEV, and ΔSrbApSrbA strains using RT-qPCR under mid-log planktonic, substratum-attached biofilm, and colony biofilm conditions.

Relative abundance of *algU* was found to be ~2.8-fold higher in the SrbA^+^ strain under planktonic conditions as observed from RT-qPCR analysis, while a ~1.3-fold lower expression level was observed in the ΔSrbA strain in comparison with the WT strain. However, in ΔSrbApSrbA, *algU* expression was ~2.2-fold higher than the WT, restoring the effect of *srbA* deletion. Under substratum-attached biofilm conditions, *algU* expression was ~2.3-fold higher in the SrbA^+^, while ~1.7-fold lower in ΔSrbA with respect to the WT strain. In ΔSrbApSrbA, *algU* expression was ~1.56-fold higher compared with the WT, effectively compensating the effect of deletion. In the colony biofilm state, *algU* expression was up-regulated by ~2.2-fold in the SrbA^+^, while a ~2.25-fold decrease was observed in ΔSrbA in comparison with the WT strain. However, in ΔSrbApSrbA, *algU* expression was ~1.71-fold higher in comparison to the WT, overcoming the effect of *srbA* deletion. Interestingly, the expression of *algU* was significantly elevated in WT cells at both the substratum-attached biofilm and colony biofilm states compared with its planktonic counterpart, by ~50.9 and ~68.12-fold, respectively. In each growth condition, the WT and ΔSrbA strains exhibited similar expression levels, when compared with their respective empty vector control strains, pEV and ΔSrbApEV (Figure 4B). It seems that the copy number of *srbA* sRNA might contribute a regulatory role in the relative abundance of *algU*, possibly through direct or indirect effect on *algU* mRNA stability. Moreover, the substantial up-regulation of *algU* in both biofilm states suggests its functional importance in biofilm formation and its maintenance.

Under planktonic conditions, the expression level of *mucA* was observed to be ~1.4-fold lower in the SrbA^+^, and ~1.15-fold higher in the ΔSrbA with respect to the WT strain. Restoration of the *srbA* deletion effect was observed in ΔSrbApSrbA, where *mucA* expression was found to be ~1.11-fold higher compared to the WT. At substratum-adhered biofilm state, *mucA* expression levels were found to be ~1.67-fold lower in SrbA^+^, and ~1.14-fold higher in ΔSrbA in comparison with the WT strain. ΔSrbApSrbA strain guided to restore the *mucA* expression level as affected by the *srbA* deletion. Under colony biofilm condition, *mucA* expression decreased ~2.9-fold in SrbA^+^ and increased by ~2.6-fold in ΔSrbA with respect to the WT strain. A lower expression level *mucA* by ~1.4-fold in ΔSrbApSrbA in comparison with the WT suggested reversal of the deletion effect of *srbA*. Interestingly, the expression of *mucA* was found to be similar in WT strain both under planktonic and substratum-attached biofilm conditions, whereas at colony biofilm state, *mucA* expression level decreased by ~28.6-fold in comparison with the planktonic WT cells (Figure 4C). Both the WT and ΔSrbA strains showed identical levels of *mucA* expression in comparison with their respective empty vector control strains, pEV and ΔSrbApEV, in each growth condition. It could be apprehended that the copy number of *srbA* might contribute in regulating *mucA* mRNA stability.

Expressional analysis revealed an increase of *rhlA* level by ~12-fold in SrbA^+^ strain and a decrease of ~4.7-fold in ΔSrbA with respect to that in the WT strain under planktonic growth condition, whereas a ~4.5-fold higher *rhlA* expression level was noted in the ΔSrbApSrbA in comparison with the WT strain, indicated restoration of the effect of *srbA* deletion. At substratum-adhered biofilm state, *rhlA* expression became ~3.3-fold increase in SrbA^+^ and ~1.7-fold decrease in ΔSrbA as compared with that in the WT strain. The complementation of *srbA* in deletion strain resulted a ~2.4-fold higher *rhlA* expression with respective to the WT strain, suggesting the recovery of the deletion effect. In the colony biofilm state, the expression of *rhlA* became ~3.0-fold increase in the SrbA^+^ strain and ~1.7-fold decrease in ΔSrbA with respect to the WT strain. The ΔSrbApSrbA strain recovered the effect of *srbA* deletion and exhibited ~2.1-fold higher compared with the WT strain. Interestingly, the expression of *rhlA* significantly increased in WT strain both under substratum-attached and colony biofilm conditions in comparison with its planktonic counterpart by ~34.0 and ~28.8-fold, respectively. At each growth condition, the WT and ΔSrbA strains showed apparently the same levels of expression compared with their respective empty vector control strains, pEV and ΔSrbApEV (Figure 4D). It is apparent from the result that the copy number of *srbA* sRNA might play a contributory function in regulating *rhlA* expression or its stability. Additionally, significant higher expression level of *rhlA* in both biofilm conditions suggests its regulatory role in biofilm development and maintenance.

In the planktonic growth condition, the *rsmA* expression levels decreased by ~2.3-fold in the SrbA^+^ and increased by ~2.5-fold in ΔSrbA in comparison with that in the WT strain. The expression of *rsmA* was found ~1.5-fold lower in ΔSrbApSrbA with respect to the WT strain, indicating reversal of deletion effect. Under substratum-attached biofilm condition, *rsmA* expression became ~1.6-fold decrease in SrbA^+^ and ~3.4-fold increase in ΔSrbA as compared with that in the WT strain. Notably, the complementation of *srbA* deletion lowered the expression level of *rsmA* by ~1.7-fold with respective to the WT strain, suggesting the recovery of the deletion effect. In the colony biofilm, the expression of *rsmA* became approximately four-fold decrease in the SrbA^+^ strain and ~4.3-fold increase in ΔSrbA compared with the WT strain. The *srbA* deletion effect on the expression level was found to be reversed in the ΔSrbApSrbA strain and showed approximately four-fold lower with respect to the WT strain. The expression of *rsmA* significantly decreased in the WT strain both under substratum-attached and colony biofilm states in comparison with its planktonic counterpart, by ~20 and ~28-fold, respectively. In each separate growth condition, the WT and ΔSrbA strains showed relatively the similar expression levels of *rsmA* compared with the corresponding empty vector control strains, pEV and ΔSrbApEV (Figure 4E). It seems that the copy number of *srbA* sRNA might contribute in the regulation of *rsmA* expression or its stability. The significant decreased expression level of *rsmA* in both biofilm states suggests its possible regulatory role in biofilm formation and maintenance.

### The role of *srbA* in regulating the translational expression of biofilm-related genes

Sequence alignment analysis indicated that *srbA* may influence the translational efficiency of certain biofilm-related genes by interacting with the ribosomal binding sites of *algU*, *mucA*, *rhlA*, and *rsmA* transcripts (Figure 4A). To explore this possibility, a translational fusion assay was performed using pUCP30T-eCFP, a plasmid containing an eCFP reporter gene. The selected gene regions, including their start codons and SD sequences, were fused at the upstream of the eCFP gene (Supplementary Figure S4-S7). These constructs were co-transformed into *E. coli* DH5α along with either the empty vector pUCP18 (EV) or pUCP18 containing *srbA*^+^ (Supplementary Figure S8 and S9). The translational efficiency of each fused gene was assessed by measuring the fluorescence intensity of cell suspensions using fluorescence microscope and a spectrofluorometer.

**Figure 5:**
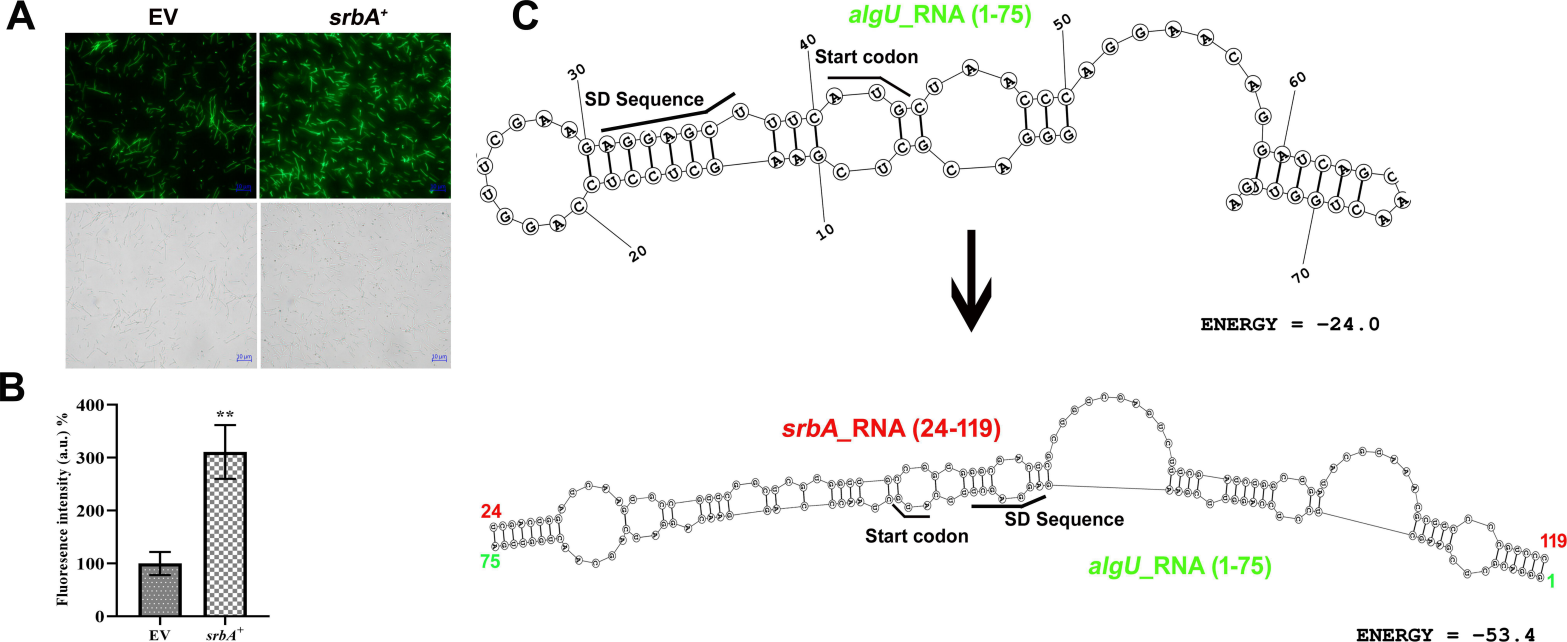
Regulatory role of *srbA* on the expression of *algU* at the translational level. (**A**) Fluorescence micrographs and bright field images of *E. coli* DH5α cells co-transformed with pUCP30T-*algU*-eCFP and either pUCP18 (EV) or pUCP18-SrbA^+^ and (**B**) fluorescence intensity of the respective cell suspensions. (**C**) Bioinformatics-derived secondary structures of *algU* mRNA (1–75 bp) alone and the duplex secondary structure of *algU* mRNA (1–75 bp), and *srbA* sRNA (24–119 bp) for visualizing the probable effect of RNA–RNA interaction on the stability of the secondary structure and *algU* mRNA translation. Values are the mean of three replications with ±SEM, and ** stands significance at *P*<0.01.

For the *algU* fusion, forward and reverse primers were designed from 43 bp upstream and 187 bp downstream of the start codon, respectively (Supplementary Figure S4). Fluorescence microscopy showed a definite increase in translation efficiency of *algU* in the presence of *srbA* (Figure 5A). A fluorescence reporter assay further confirmed this finding that showed a ~3.1-fold increase in fluorescence intensity when pUCP30T-*algU*-eCFP was co-transformed with pUCP18-*srbA*^+^ in comparison with the empty vector control (Figure 5B). The results suggest that the *srbA* sRNA have regulatory role on the expression of the *algU* transcript possibly by facilitating ribosome binding. Bioinformatics analysis was further done to unveil the possible RNA–RNA interactions and the stability of the secondary structures involved. The analysis predicted that the ribosome binding site, SD sequence, and start codon of *algU* formed a highly structured, intramolecular base-paired region, which might hinder ribosome binding and proper translation initiation. However, binding of *srbA* sRNA with the *algU* RNA resulted RNA duplex structure, which was appeared to be more stable, with the SD sequence and start codon positioned favorably for ribosome binding. This structural rearrangement seems to enhance the translational initiation of *algU* by the *srbA* sRNA (Figure 5C).

**Figure 6:**
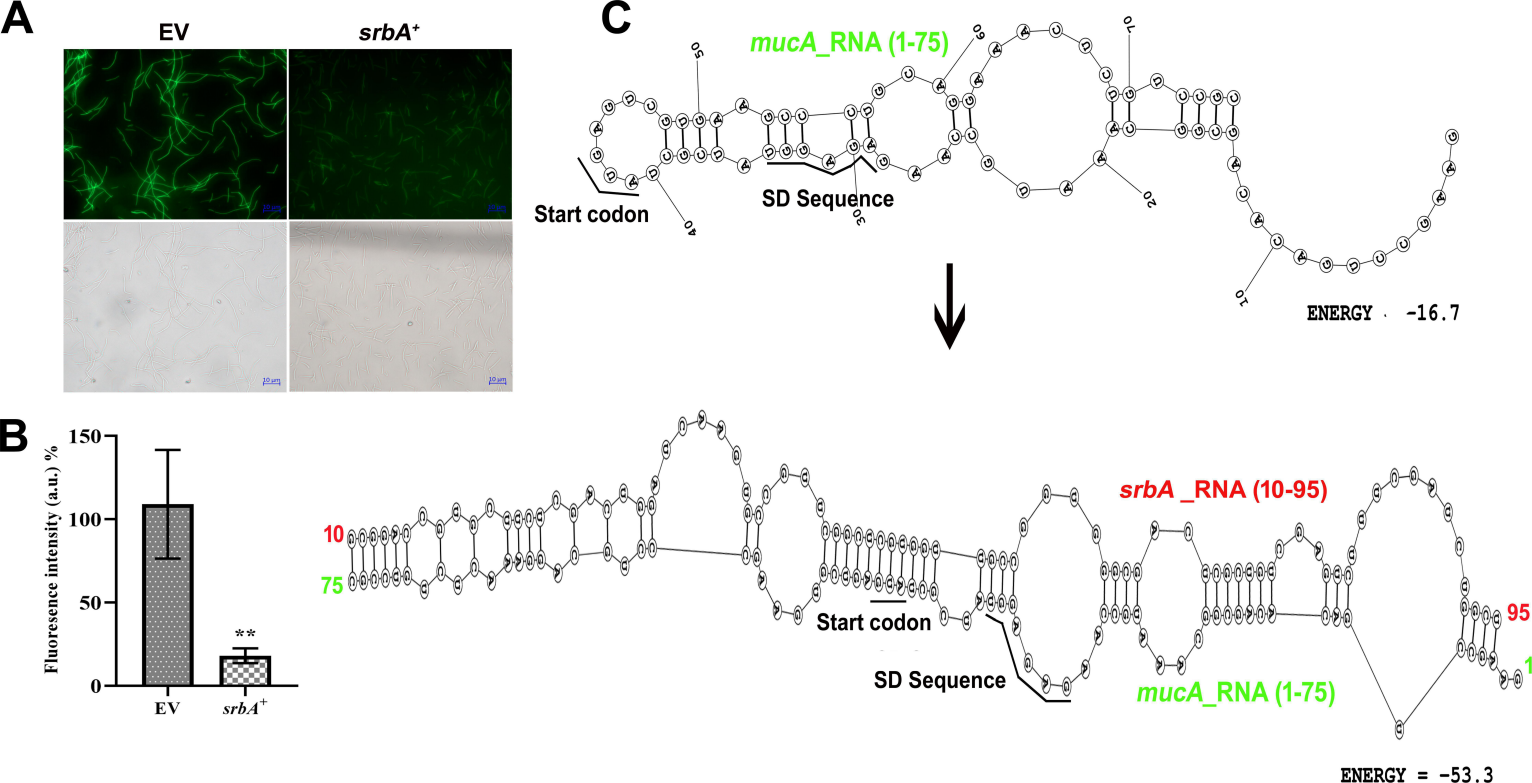
Regulatory role of *srbA* on the expression of *mucA* at the translational level. (**A**) Fluorescence micrographs and bright field images of *E. coli* DH5α cells co-transformed with pUCP30T-*mucA*-eCFP and either pUCP18 (EV) or pUCP18-SrbA^+^, and (**B**) fluorescence intensity of the respective cell suspensions. (**C**) Bioinformatics-derived secondary structures of *mucA* mRNA (1–75 bp) alone and the duplex secondary structure of *mucA* mRNA (1–75 bp), and *srbA* sRNA (10–95 bp) for visualizing the probable effect of RNA–RNA interaction on the stability of the secondary structure and *mucA* mRNA translation. Values are the mean of three replications with ±SEM, and ** stands significance at *P*<0.01.

For the *mucA* fusion, forward and reverse primers were designed 41 bp upstream and 93 bp downstream of the start codon, respectively (Supplementary Figure S7). Fluorescence microscopy showed a notable reduction in the translation efficiency of *mucA* when *srbA* sRNA was present (Figure 6A). This was further supported by the fluorescence reporter assay, which demonstrated a six-fold reduction in fluorescence intensity when pUCP30T-*mucA*-eCFP was co-transformed with pUCP18-*srbA*^+^ in comparison with the empty vector control (Figure 6B). Results suggest that *srbA* sRNA might directly interact with the *mucA* transcript, thus inhibiting ribosome binding, and consequently lowering *mucA* translation. Further bioinformatics analysis examined the potential RNA–RNA interactions and secondary structure stability of *mucA*. The analysis revealed that the ribosome binding site, SD sequence, and start codon of *mucA* were properly aligned for ribosome binding and translation initiation under normal conditions. However, when *srbA* sRNA binds to *mucA* RNA, the formation of a more stable RNA duplex structure was observed (Figure 6C). The results suggest that *srbA* sRNA regulates *mucA* transcript expression possibly by inhibiting ribosome binding and thereby reducing its translation.

**Figure 7:**
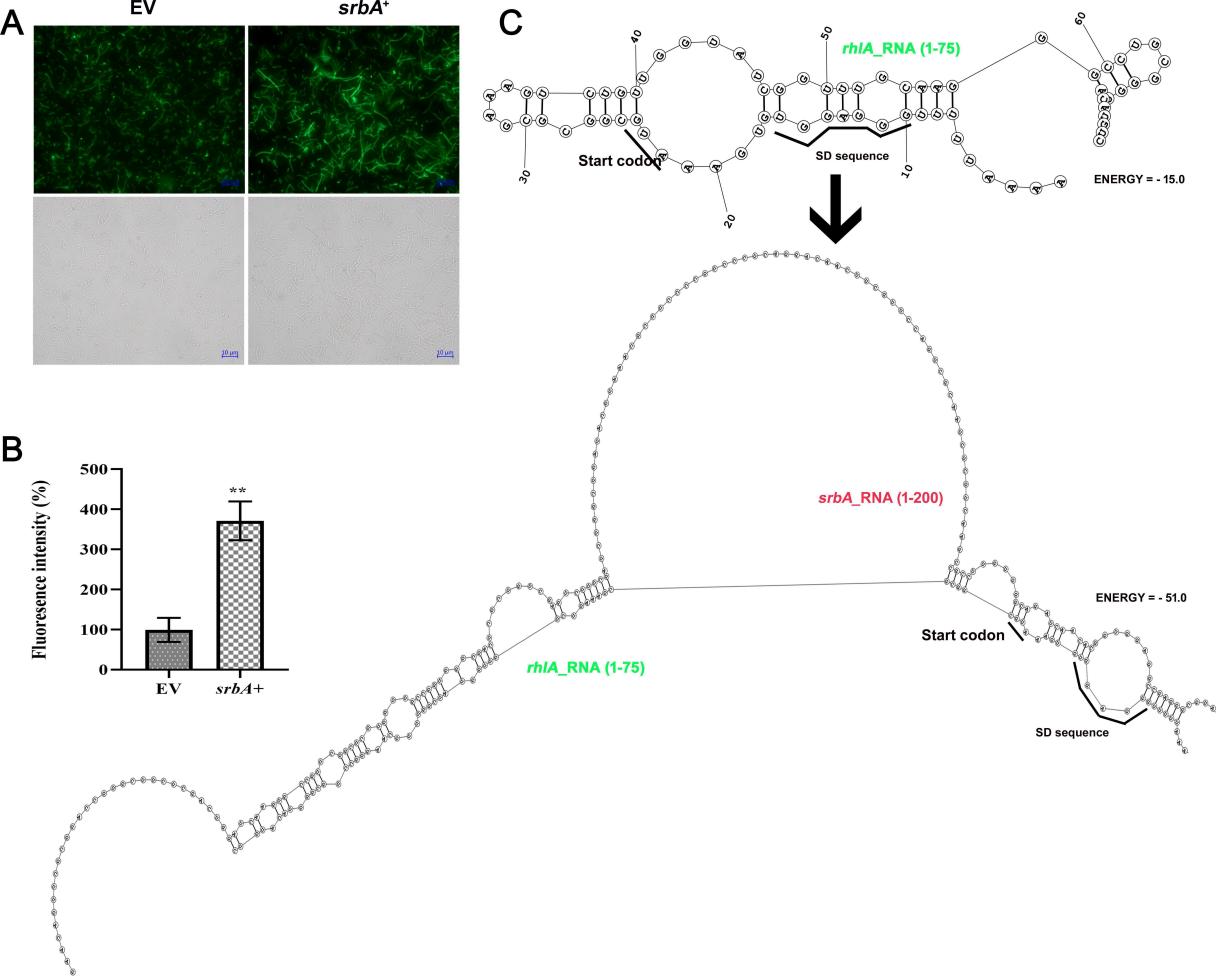
Regulatory role of *srbA* on the expression of *rhlA* at the translational level. (**A**) Fluorescence micrographs and bright field images of *E. coli* DH5α cells co-transformed with pUCP30T-*rhlA*-eCFP and either pUCP18 (EV) or pUCP18-SrbA^+^ and (**B**) fluorescence intensity of the respective cell suspensions. (**C**) Bioinformatics-derived secondary structures of *rhlA* mRNA (1–75 bp) alone and the duplex secondary structure of *rhlA* mRNA (1–75 bp), and *srbA* sRNA (1–85 bp) for visualizing the probable effect of RNA–RNA interaction on the stability of the secondary structure and *rhlA* mRNA translation. Values are the mean of three replications with ±SEM, and ** stands significance at *P*<0.01.

For the *rhlA* fusion, primers were designed from 27 bp upstream and 103 bp downstream of the start codon (Supplementary Figure S5). Fluorescence microscopy showed a notable increase in the translation efficiency of *rhlA* when *srbA* sRNA was present (Figure 7A). This observation was further confirmed by fluorescence reporter assay, which showed a ~3.7-fold increase in fluorescence intensity when pUCP30T-*rhlA*-eCFP was co-transformed with pUCP18-*srbA*^+^ in comparison with the empty vector control (Figure 7B). Bioinformatics analysis was explored to find the possible RNA–RNA interactions and the stability of the secondary structures involved. The analysis predicted that the ribosome binding site, SD sequence, and start codon of *rhlA* formed a highly structured region with intramolecular base pairing, which could hinder ribosome binding and translation initiation. However, when *srbA* sRNA binds to *rhlA* RNA, a more stable RNA duplex structure forms, positioning the SD sequence and start codon in a way that facilitates ribosome binding (Figure 7C). The results indicate that *srbA* sRNA enhance the expression of the *rhlA* transcript likely by promoting ribosome binding and increasing its translation efficiency.

**Figure 8:**
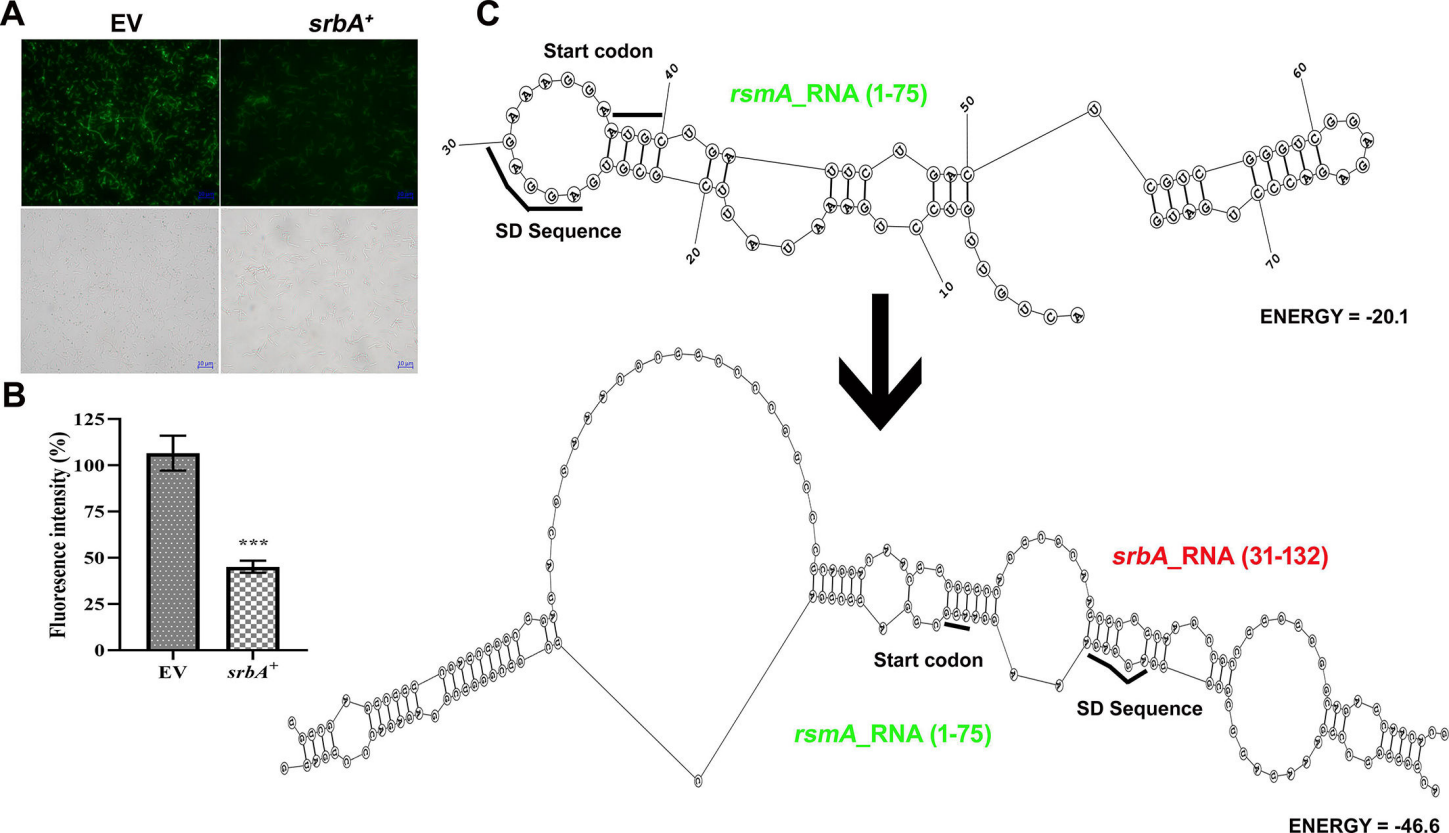
Regulatory role of *srbA* on the expression of *rsmA* at the translational level. (**A**) Fluorescence micrographs and bright field images of *E. coli* DH5α cells co-transformed with pUCP30T-*rsmA*-eCFP and either pUCP18 (EV) or pUCP18-SrbA^+^ and (**B**) fluorescence intensity of the respective cell suspensions. (**C**) Bioinformatics-derived secondary structures of *rsmA* mRNA (1–75 bp) alone and the duplex secondary structure of *rsmA* mRNA (1–75 bp), and *srbA* sRNA (31–132 bp) for visualizing the probable effect of RNA–RNA interaction on the stability of the secondary structure and *rsmA* mRNA translation. Values are the mean of three replications with ±SEM, and *** stands significance at *P* < 0.001.

For the *rsmA* fusion, forward and reverse primers were designed from 41 bp upstream and 91 bp downstream of the start codon, respectively (Supplementary Figure S6). Fluorescence microscopy indicated a substantial decrease in the translation efficiency of *rsmA* when *srbA* sRNA was present (Figure 8A). This was further validated by fluorescence reporter assay, which showed a ~2.4-fold reduction in fluorescence intensity when pUCP30T-*rsmA*-eCFP was co-transformed with pUCP18-*srbA*^+^ in comparison with the empty vector control (Figure 8B). Further bioinformatics analysis was investigated to find possible RNA–RNA interactions and secondary structure stability for *rsmA*. The analysis revealed that, under normal conditions, the ribosome binding site, SD sequence, and start codon of *rsmA* are well-positioned for ribosome binding and translation initiation. However, when *srbA* sRNA binds to *rsmA* RNA, a more stable RNA duplex structure forms, negating ribosome binding through intermolecular base pairing, which likely results to the decrease of *rsmA* translation in the presence of *srbA* sRNA (Figure 8C). The finding suggests that *srbA* sRNA may down-regulate *rsmA* transcript expression probably by preventing ribosome binding, leading to reduced translation.

## Discussion

With the alarming emergence of multidrug-resistant *P. aeruginosa* strains and its versatile adaptability, understanding the regulatory network that allows this bacterium to adapt and thrive in diverse environments is crucial for combating infections. Approximately one tenth of *P. aeruginosa* genome is dedicated for encoding different transcriptional modulators, along with abundant sRNAs dispersed throughout its genome. In many cases, sRNAs exert their influence through base-pairing with target mRNAs, thus modulate the expressions of the genes. This interaction may occur either in untranslated regulatory regions or coding regions of the mRNA, thereby activating or repressing the translation [43–45]. The regulatory role of sRNAs in controlling stress adaptation and virulence is well established in *P. aeruginosa* [32,33,46–48]. A sophisticated regulatory machinery, operating via transcriptional, post-transcriptional, and post-translational courses in response to the environmental and host-derived signals through the QS system, modulates adaptability, biofilm formation, motility, and pathogenicity of *P. aeruginosa* [5,8,10,49–52]. Understanding these genetic regulatory mechanisms is essential for addressing the molecular basis of pathogenicity and developing strategies to combat the opportunistic infections caused by *P. aeruginosa*.

The *srbA* sRNA was earlier documented to be up-regulated at the stationary growth phase and biofilm state of *P. aeruginosa* PA14, though its exact molecular regulatory role in biofilm formation remained unclear yet [37]. In this study, *srbA* was found to be up-regulated during biofilm and colony biofilm growth phases in *P. aeruginosa* PAO1 strains, suggesting its involvement in the development of biofilm. The differences in *srbA* expression levels observed among the planktonic, substratum-attached biofilm, and colony biofilm states in either SrbA^+^ or ΔSrbApSrbA are intriguing, even though the *srbA* is overexpressed from the same plasmid (Figure 1B). Generally, various factors collectively affect the overall expression profile of genes, be it on bacterial chromosomes or plasmids, depending on their growth conditions. Alteration in critical or limiting factors under different growth conditions may regulate the copy number of *srbA* through various mechanisms, such as plasmid copy number, promoter activity, and RNA stability. The regulatory role of *srbA* in biofilm development was further investigated using *srbA* deletion and overexpression strains. The results revealed that *srbA* overexpression enhanced biofilm formation, while its deletion reduced biofilm development. The study of colony morphology revealed that the SrbA^+^ colonies exhibited a higher density of EPS, whereas the ∆SrbA colonies displayed a distinct morphology, characterized by a smaller colony diameter and reduced quantity of EPS (Figure 2E). This observation aligns with earlier findings on the role of *srbA* in regulating motility in *P. aeruginosa* [33]. Earlier studies on *MacS* sRNA of *E. coli* suggested its regulatory role on biofilm formation by controlling bacterial motility and polysaccharide production [50]. Similarly, in *P. aeruginosa*, *RsmZ* and *RsmY* sRNAs stimulate biofilm formation by inhibiting RsmA activity [34,53], while *ErsA* regulates biofilm formation by modulating AmrZ at the post-transcriptional level [46]. Additionally, *PA0730.1* sRNA has been reported to regulate biofilm formation through its control over the *mucA* and *rpoS* genes in *P. aeruginosa* [32]. These findings suggest that *srbA* might also target specific genes to play diverse roles in biofilm development.

AlgU is a critical σ-factor that regulates the expression of numerous genes involved in survival of *P. aeruginosa* under diverse environmental situations and the production of various virulence factors [54]. AlgU has an autoregulatory function through its interaction with anti-sigma factor MucA and controls the production of biofilm architectural components, particularly alginate [55]. MucA is responsible for regulating the mucoid phenotype often associated with chronic lung infections caused by *P. aeruginosa* [21,56,57]. Both *algU* and *mucA* are components of the same operon (algUmucABCD), which is transcribed from shared promoters (P1–P5) under the regulatory control of the sigma factor AlgT/U [58,59]. The differential effects of *srbA* on the transcript levels of *algU* and *mucA* suggest that its regulatory role is more likely to occur at a post-transcriptional level, rather than at the transcriptional level. It seems that the observed differences may arise from selective modulation of mRNA stability or processing by *srbA*. Overexpression of *srbA* resulted in increased expression of *algU*, which may explain the enhanced biofilm formation observed in *srbA* overexpression strains. Furthermore, a significant reduction in *mucA* translation was detected in strains with higher *srbA* copy numbers, leading to increased levels of active AlgU. Thus, the combined positive regulation of *algU* and negative regulation of *mucA* by *srbA* likely contributes to its role in biofilm formation by *P. aeruginosa*. Furthermore, an increase in AlgU levels coupled with a decrease in MucA levels is expected to lead to enhanced mucoid phenotype, which correlates with the findings from the alginate production and colony morphological nature (Figure 2D and E).

In *P. aeruginosa*, rhamnolipid production is crucial for maintaining biofilm structure, as it modulates the biofilm surface properties and facilitates microcolony formation [8]. The enzyme RhlA, which plays a key role in rhamnolipid biosynthesis, is modulated by the Rhl system [24]. Overexpression of *srbA* leads to increased expression of *rhlA* at both the transcriptional and translational levels, which likely explains the enhanced biofilm formation observed in *srbA* overexpressing strains. Additionally, SrbA^+^ strains were found to produce higher levels of rhamnolipids than WT *P. aeruginosa*, both in nutrient-rich and nutrient-limited conditions [33]. Increased rhamnolipid production also correlates with the previous findings on enhanced swarming motility of SrbA^+^ strain [33].

RsmA, a critical regulator in *P. aeruginosa*, is part of the CsrA family of RNA-binding proteins and has been shown to control virulence, motility, biofilm formation, and metabolism by interacting with target mRNAs [27–29]. RsmA inhibits biofilm formation by suppressing the production of EPS such as Psl and Pel, key components of the biofilm matrix, and modulates flagellar motility, promoting bacterial dispersal [29,60]. In *srbA* overexpressing strains, *rsmA* expression was reduced at both the transcriptional and translational levels, contributing to the enhanced biofilm formation in these strains. Moreover, changes in *rsmA* levels could affect virulence traits, which corroborates with previous observations on the role of *srbA* in regulating the synthesis of various virulence factors in *P. aeruginosa* [33].

**Figure 9:**
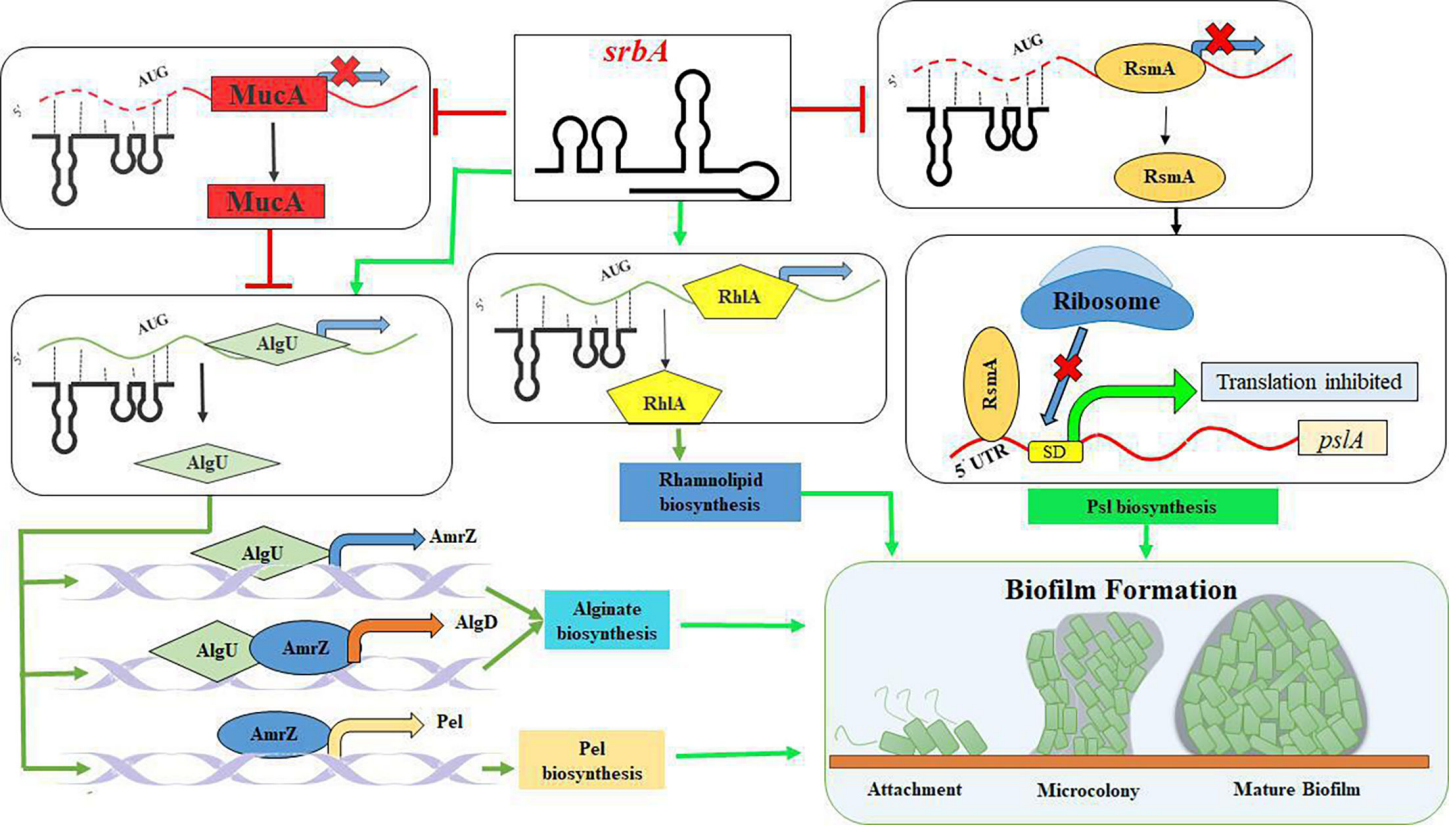
The possible molecular targets of *srbA* sRNA for regulating QS-controlled biofilm formation in *P. aeruginosa*. QS, quorum sensing; sRNA, small RNA.

These findings have unveiled the pivotal role *srbA* as a modulator of various genes, such as *algU*, *mucA*, *rhlA*, and *rsmA*, involved in regulatory network for biofilm development in *P. aeruginosa* (Figure 9). However, to verify a direct interaction, nucleotide substitutions should be performed both in the sRNA and the predicted mRNA interaction region. The functional intricacy of sRNA is seemed to be greater than previously thought about, and it warrants in-depth genetic and structural analyses to understand such regulatory networks controlled by *srbA*, especially in host-pathogen interactions. The outcome of this study suggests *srbA* as a promising drug target and to develop oligonucleotide-based therapeutic strategies for combating the threat of multidrug-resistant *P. aeruginosa*.

## Methods

### Bacterial strains, plasmid, and growth conditions

*Pseudomonas aeruginosa* PAO1 (ATCC 15692) used as the experimental model organism in this study. For cloning and translational fusion experiments, *Escherichia coli* DH5α was used. Details of the plasmids and bacterial strains used in this study are given in Supplementary Table S1 and S2, respectively. The *srbA* overexpression (SrbA^+^), *srbA* deletion (ΔSrbA), and plasmid-mediated complementation of *srbA* deletion (ΔSrbApSrbA) strains, and their respective control, empty vector (pEV), WT, and complementation of deletion strain by empty vector (ΔSrbApEV) strains were constructed earlier [33] and were used for studying the effect of *srbA* sRNA on biofilm development in *P. aeruginosa* PAO1. For the planktonic culture, cells were grown in the LB (Luria Bertani) broth for 3 h in 37°C at 200 rpm shaking condition or till OD_600_ of 0.8. For developing substratum-attached biofilm, cells were grown in LB broth and kept at 37°C under static condition for 24 h. The colony biofilm was prepared in LB agar plate, where 5 µl of mid-log phase culture was spot inoculated and was kept for 48 h at 37°C. For the translation fusion analysis, the *E. coli* cells were grown in the LB broth. Selective antibiotics were supplemented in the specified media as per the experimental requirements and the strains used. The antibiotics were used in this study were carbenicillin 150 µg/ml, gentamycin 100 µg/ml, tetracycline 100 µg/ml for strains of *P. aeruginosa*, and ampicillin 50 µg/ml, gentamycin 50 µg/ml for the strains of *E. coli*.

### Strain construction

The strains pEV, SrbA^+^, ΔSrbA, ΔSrbApEV, and ΔSrbApSrbA were earlier constructed in the laboratory and were used in this study [33]. For confocal microscopy, the eCFP reporter-based plasmid pUCP30T-eCFP was electroporated into the WT PAO1 strain, along with pEV, SrbA^+^, ΔSrbA, and ΔSrbApSrbA strains. For the translational fusion assay, *srbA* was inserted into the pUCP18 plasmid [33], and its target mRNA was cloned into the pUCP30T-eCFP plasmid containing an eCFP reporter gene [61]. Specified regions of the selected gene, including start codons and SD sequences, were fused upstream of the eCFP gene. For *mucA* translation fusion, the pUCP30T-*mucA*-eCFP construct was used [32]. The pUCP30T-*algU*-eCFP construct was developed using forward and reverse primers designed from the transcription initiation site and downstream regions of the *algU* gene, respectively. Similarly, pUCP30T-*rhlA*-eCFP and pUCP30T-*rsmA*-eCFP were constructed with corresponding primers for the *rhlA* and *rsmA* genes. All primers used for the strain construction are presented in Supplementary Table S3.

### Biofilm quantification

Biofilm quantification using the crystal violet assay was conducted as described earlier [62]. Bacterial cultures (OD_600_ = 0.4) from mid-exponential phase of WT, pEV, SrbA^+^, ΔSrbA, and ΔSrbApSrbA strains were inoculated in a 96-well microtiter plate and incubated at 37°C without any shaking. After incubation, the culture broth was carefully pipetted out, and the adhered biofilm was gently rinsed with sterile PBS. Biofilms were then fixed with methanol, air-dried, and were stained with 0.1% Hucker crystal violet. Excess dye was removed, and the crystal violet retained by the biofilm was dissolved with 33% glacial acetic acid. The absorbance was measured at 570 nm using a microtiter plate reader (iMARK, Bio-Rad, Japan).

### MTT assay for cell viability

Cell viability in biofilms was determined by the MTT assay [63]. The biofilms formed by WT, pEV, SrbA^+^, ΔSrbA, and ΔSrbApSrbA in the 96-well microtiter plate were washed with PBS, and 200 μl of LB containing 0.5 mg/ml MTT reagent was poured to each well. After incubation of 2 h at 37°C in the dark, the formazan crystals formed were dissolved with DMSO, and the A_570_ was measured by the microtiter plate reader.

### Congo red binding assay

The amount of EPS in the biofilms was quantified by Congo red binding assay [64]. Biofilms formed by WT, pEV, SrbA^+^, ΔSrbA, and ΔSrbApSrbA strains in a 96-well plate were stained with 1% Congo red, incubated in the dark for 30 min, and were then washed with PBS. Bound dye was dissolved in DMSO, and absorbance was recorded at 490 nm.

### Alginate assay

The alginate production was determined following the method described earlier [65]. Cells of WT, pEV, SrbA^+^, ΔSrbA, and ΔSrbApSrbA from the substratum-attached biofilm were suspended in 500 μl PBS. The suspension was then mixed with 500 μl of 1 M NaCl and was vortexed to extract the alginate bound to the cell surface. The resulting mixture was then centrifuged at 8000 × *g* for 20 min. The supernatant was mixed with cetylpyridinium chloride (2% w/v) and was kept overnight at 4°C. The alginate–cetylpyridinium chloride complex was subsequently collected by centrifugation at 8000 × *g* for 10 min at 4°C. The supernatant was discarded, and the pellet was dissolved in 500 μL of chilled isopropanol. This mixture was then centrifuged again at 8000 × *g* for 10 min at 4°C. The pellet was resuspended in 1 M NaCl and was kept overnight at 4°C. The alginate content was quantified using the carbazole method [66].

### Colony morphology in Congo red agar plate

For colony morphology, mid-log phase cultures of WT, pEV, SrbA^+^, ΔSrbA, and ΔSrbApSrbA were point inoculated onto tryptone agar plates having Congo red and Coomassie brilliant blue. After an incubation of 24 h at 37°C, morphological features of the colonies were visualized under a stereo microscope (Zoomstar-II, Dewinter).

### Confocal and scanning electron microscopy

CLSM and SEM were used to observe biofilm architecture. For both microscopic studies, WT, pEV, SrbA^+^, and ΔSrbA strains transformed with pUCP30T-eCFP were grown on coverslips in a 24-well plate and were incubated for 24 h. Biofilms were washed to remove unadhered cells, fixed with 2.5% glutaraldehyde, then again washed with PBS, and were air-dried. For CSLM, the images were captured using a confocal microscope (Zeiss LSM 800, Carl Zeiss, Germany) with a 434 nm excitation and 477 nm emission wavelengths to detect eCFP fluorescence in the cells embedded within the biofilm matrices. COMSTAT analysis was performed for measuring the average bio-volume and thickness of the biofilm images. For SEM, the fixed-dried biofilm on the coverslips was dehydrated with a gradient of ethanol and was then dehydrated thrice with absolute ethanol for 10 min each. After platinum coating, the images were captured by a SEM (Evo LS10, Carl Zeiss, Germany).

### Translational fusions assay

The involvement of *srbA* on the translational efficiency of selected genes was assessed using translational fusion assay by transforming *E. coli* DH5α with eCFP reporter plasmid constructs [pUCP30T-*algU*(-43 to +187)-eCFP, pUCP30T-*mucA*(-41 to +91)-eCFP, pUCP30T-*rhlA*(-27 to +103)-eCFP, pUCP30T-*rsmA*(-41 to +91)-eCFP] co-transformed with SrbA^+^ or EV strains [32,33]. Cells from mid-log phase culture of each strain were inoculated to LB and grown at 37°C to attain an OD_600_ of ~0.2. Isopropyl β-d-1-thiogalactopyranoside (IPTG) of a final concentration of 50 µg/ml was added to the growing culture and was kept further for 2 h. The cell pellets were washed using PBS by centrifugation, and the cell suspension in PBS was put on glass slide, and finally the fluorescence was measured using fluorescence microscope (Axio Vert.A1 FL-LED, Carl Zeiss, Germany). The fluorescence intensity of eCFP in the cell suspensions of each strain was determined using an excitation and emission wavelength of 434 nm and 477 nm, respectively, by a fluorescence spectrophotometer (F-7100, Hitachi, Japan).

### Expression study by real-time PCR

Total RNA from biofilm and planktonic cultures was extracted by the TRIzol MAX Bacterial RNA isolation kit (Ambion Lifetechnology, U.S.A.) and was digested with DNase to remove any DNA contamination. The cDNA was synthesized using 2 µg of RNA and random hexamers, following the manufacturer’s manual (Applied Biosystems, U.S.A.). The real-time PCR (RT-qPCR) was carried out using a thermal cycler (StepOne Real-Time PCR System, Applied Biosystem, U.S.A.). A reaction mixture of 20 μl was prepared using 10 μl of Power UP™ SYBR™ Green Master Mix (Applied Biosystems, U.S.A.), 0.4 μl both of forward and reverse primers (20 μM), 7.2 μL nuclease-free water, and 2 μl cDNA. The cycle of reaction was set with an initial holding stage at 50°C for 2 min and then at 95°C for 2 min, denaturation was done at 95°C for 15 sec. Annealing temperature and extension time were set according to the Tm of the respective primer and amplicon size. Each RT-qPCR study was performed in triplicate. The level of quantitative expression for each selected gene was analyzed comparing that in the respective control set, and *rpoD* was considered as the reference house-keeping gene for the normalization. The primers used for this RT-qPCR study are listed in Supplementary Table S3.

### Bioinformatics analysis

*Pseudomonas* PAO1 gene sequences were obtained from the *Pseudomonas* genome sequence database [38]. Complementary base-pair analysis of *srbA* with biofilm regulatory transcripts was carried out using IntaRNA 2.0 [42], using default set up (Supplementary Figure S1). The RNA secondary structure prediction was done using RNAStructure 6.0.1 [67].

### Statistical analysis

Data were analyzed for all experiments by descriptive statistics and one-way ANOVA using GraphPad Prism version 9.0. All experiments were done in triplicate, and results are showed as the mean ± SEM.

## Supplementary material

Online supplementary figures and tables

## Data Availability

All data generated or analyzed during this study are included in this published article (and its Supporting information files).

## Abbreviations

CSLM: confocal scanning laser microscopy
QS: quorum sensing
SEM: scanning electron microscopy
WT: wildtype
sRNAs: small RNAs
