# Supplementary material for: Unveiling the role of srbA sRNA in biofilm formation by regulating algU, mucA, rhlA, and rsmA in Pseudomonas aeruginosa
Source: Biochem J. 2025 May 23;482(11):621–37. doi: 10.1042/BCJ20240650 (PMC12203948; doi:10.1042/BCJ20240650)
Supplement: Online supplementary figures and tables [file BCJ-482-11-BCJ20240650-s001.docx]

**Supplementary data**

**Title**: Unveiling the role of *srbA* sRNA in biofilm formation by regulating *algU*, *mucA*, *rhlA*, and *rsmA* in *Pseudomonas aeruginosa*

**Authors**: Piyali Saha, Samir Kumar Mukherjee, Sk Tofajjen Hossain*

**Affiliation**: Department of Microbiology, University of Kalyani, Kalyani 741235, India

***Corresponding Author**:

Dr. Sk Tofajjen Hossain

Department of Microbiology, University of Kalyani, Kalyani 741235, India

Email. [tofajjenmicro22@klyuniv.ac.in](mailto:tofajjenmicro22@klyuniv.ac.in); [sktofajjen.hossain@gmail.com](mailto:sktofajjen.hossain@gmail.com)

Phone: +91-7001902413

**Running title:** The role of *SrbA* in biofilm formation in *P. aeruginosa*

**Table S1.** Plasmids used in this study

| **Name of the plasmids** | **Description** | **Reference** |
| --- | --- | --- |
| pUCP18 | An *E. coli P. aeruginosa* shuttle vector | 3 |
| pUCP18-*srbA* | *srbA* overexpression plasmid | 1 |
| pACRISPR | A sgRNA expression plasmid for mutant preparation in *P. aeruginosa* | 4 |
| pCasPA | Cas9 expression plasmid under λ-Red system for *P. aeruginosa*, | 4 |
| pACRISPRNN*srbA* | *srbA* spacer inserted pACRISPR plasmid | 1 |
| pUCP30T-eCFP | Broad host range ECFP reporter plasmid | 5 |
| pUCP30T-*algU*-eCFP | *algU* gene along with SD-sequence cloned in the pUCP30T-eCFP plasmid (43bp-Start codon-189bp) | In this study |
| pUCP30T-*mucA*-eCFP | *mucA* gene along with SD-sequence cloned in the pUCP30T-eCFP plasmid (41bp-Start codon-93bp) | 2 |
| pUCP30T-*rhlA*-eCFP | *rhlA* gene along with SD-sequence cloned in the pUCP30T-eCFP plasmid (27bp-Start codon-105bp) | In this study |
| pUCP30T-*rsmA*-eCFP | *rsmA* gene along with SD-sequence cloned in the pUCP30T-eCFP plasmid (41bp-Start codon-93bp) | In this study |

**Table S2.** Bacterial strains used in this study

| **Strains** | **Description** | **Reference** |
| --- | --- | --- |
| *P*. *aeruginosa* PAO1 (ATCC 15692) | Used as the model organism | Lab stock |
| *E*. *coli* DH5α | For cloning and translation fusion assay | Lab stock |
| SrbA^+^ | pUCP18-*srbA* plasmid in PAO1 strain | 1 |
| pEV | pUCP18 plasmid in PAO1 strain | 1 |
| ΔSrbA | *srbA* region deleted from PAO1 genome | 1 |
| ΔSrbApEV | pUCP18 plasmid in ΔSrbA strain | 1 |
| ΔSrbApSrbA | pUCP18-*srbA* plasmid in ΔSrbA strain | 1 |

**Table S3.** Primers used in this study

| **NAME** | **PRIMER SEQUENCE (5´-3´)** | **Reference** |
| --- | --- | --- |
| SrbA qF | ACTCGCTGTCGAGTCTTTCG | 1 |
| SrbA qR | TGCGACTGGAACGAAGTTGT | 1 |
| AlgU-qF | ATACTGGGATTGATCGTGCG | This study |
| AlgU-qR | TGTTGATGGCGATCCGATAC | This study |
| MucA-qF | TGTCCGCTGTGATGGATAACG | This study |
| MucA-qR | ATATCCAGCTTCGGCAGGGTAG | This study |
| RhlA-qF | GCCGAACATTTCAACGTGG | This study |
| RhlA-qR | TGATTGACCTCGAAGCGC | This study |
| RsmA-qF | AGAGACCCTGATGGTAGGTG | This study |
| RsmA-qR | TGGTAAATTTCCTCCCGGTG | This study |
| AlgU-TF-F | AT**TCTAGA**GCTGGGACGCTCGAAGCT | This study |
| AlgU-TF-R  mucA_TF F  mucA_TF R | CAT**GCATGC**CGAGCGCACGGTATGCCT  GC**TCTAGA**AGAAGCCTGACACAGCGG  ATT**GCATGC**CGCAAGCTGCGAGCACC | This study  2  2 |
| RhlA-TF-F | GG**TCTAGA**TGTTCGAAAATTTTTGGGAGG | This study |
| RhlA-TF-R | TTAG**GCATGC**CGTTGACCAGCATCACCGT | This study |
| RsmA-TF-F | TG**TCTAGA**TGCAGACTGTTGTCCTGAAA | This study |
| RsmA-TF-R | AATTG**GCATGC**GCACCTGGTTCCCTTTGAC | This study |

**Figure S1**

**
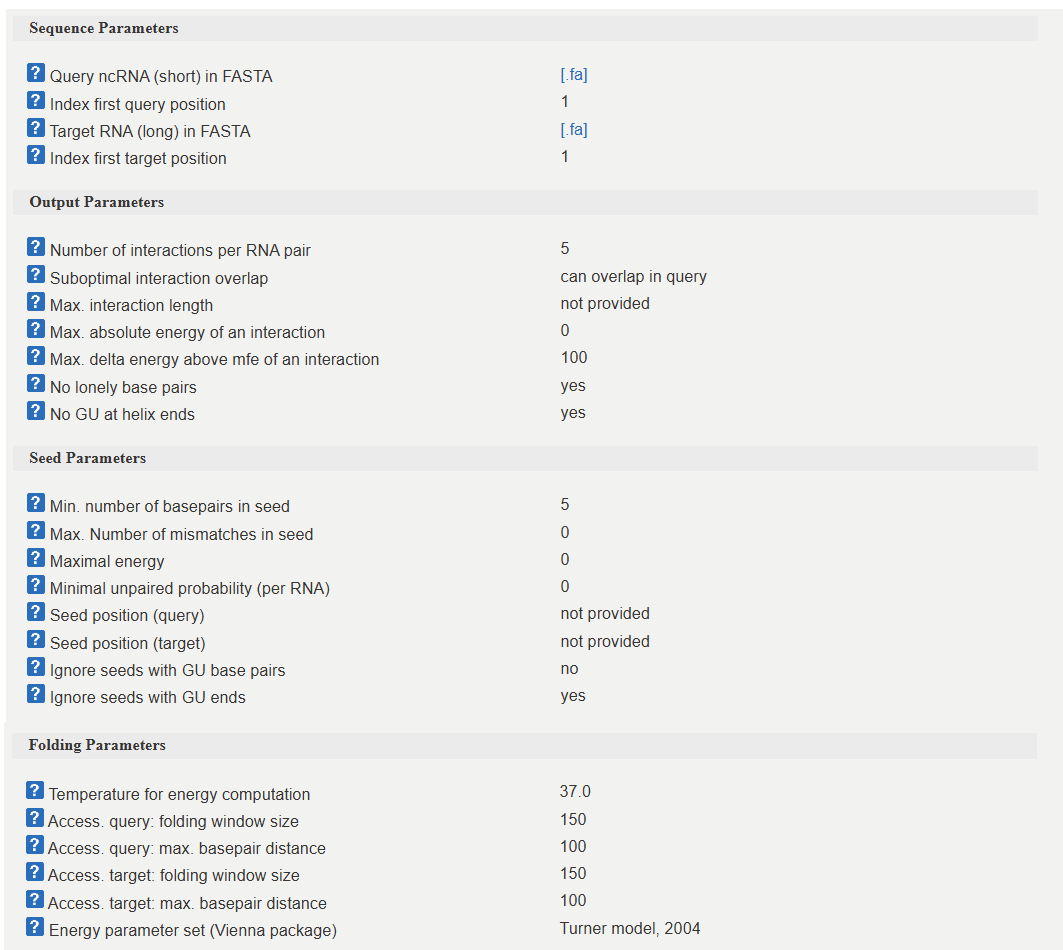
**

**Figure S1.** Default settings in the IntaRNA tool for the RNA-RNA interactions analysis.

**Figure S2**

**
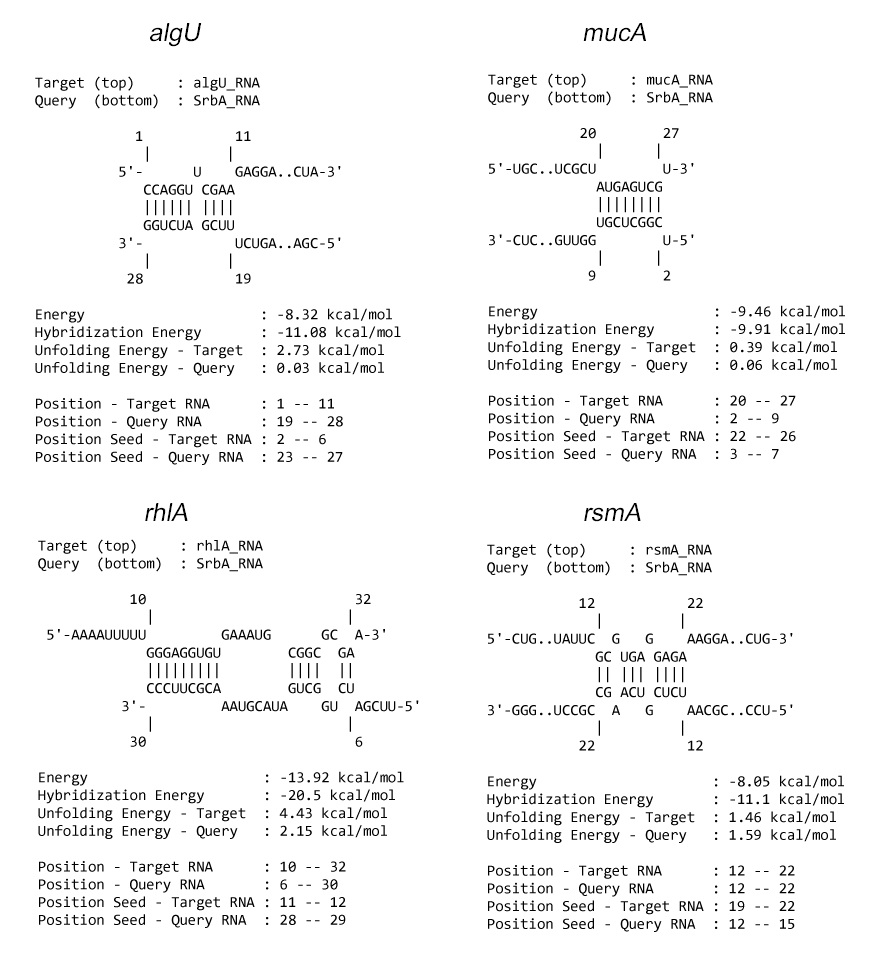
**

**Figure S2.** The output results as obtained during RNA-RNA interactions study for *srbA* sRNA with *algU*, *mucA*, *rhlA*, and *rsmA* mRNA using IntaRNA 2.0.

**Figure S3**

**
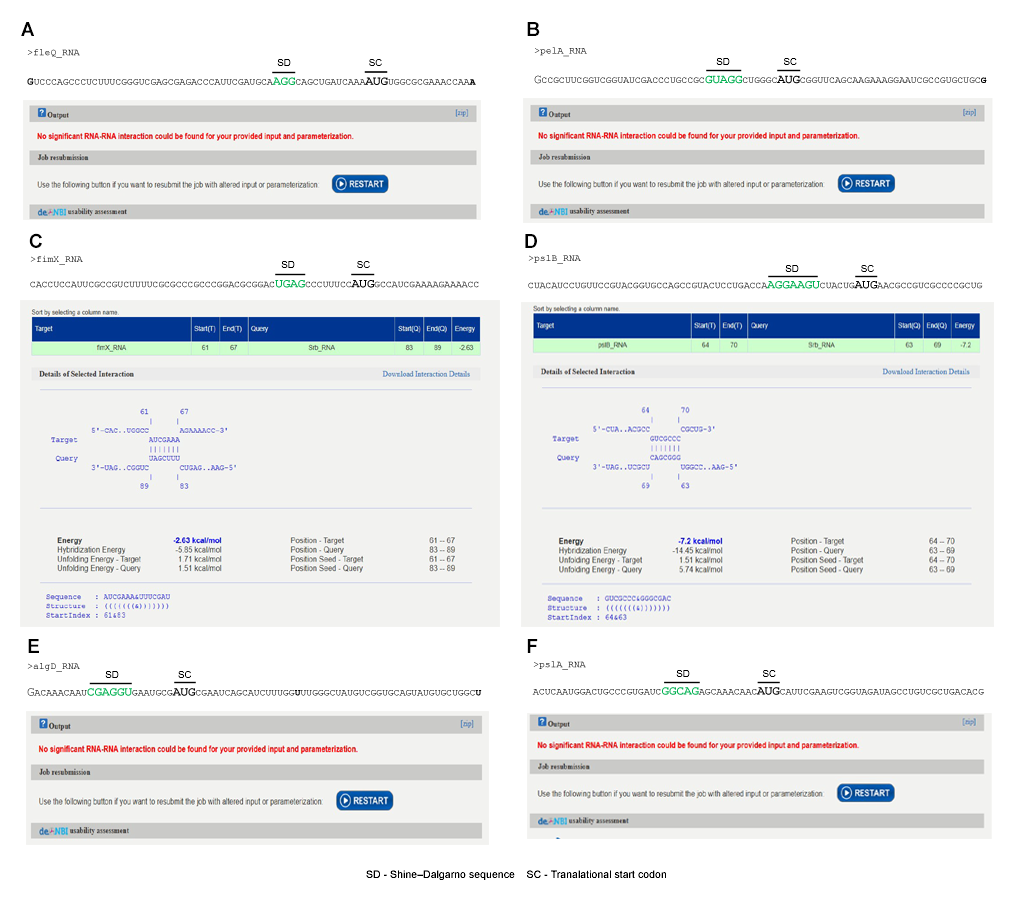
**

**Figure S3.** The mRNA sequence (75 bp) and the corresponding output results obtained from the RNA-RNA interaction study of *srbA* sRNA with the genes those were not considered for further analysis - (A) *fleQ*; (B) *pelA*; (C) *fimX*; (D) *pslB*; (E) *algD*; (F) *pslA*.

**Figure S4**

**
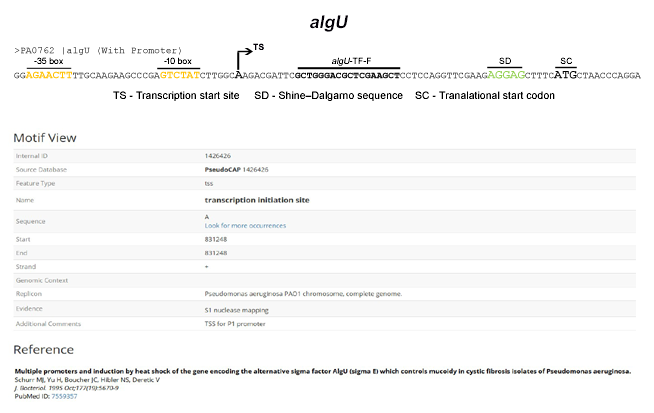
**

**Figure S4.** Primer positions in the 5'-UTR region relative to the transcription start site, Shine-Dalgarno sequence, and translational start codon of *algU* for the translational fusion study. The transcription start site was annotated based on the *Pseudomonas* genome database [6], with the specific reference source.

**Figure S5**

**
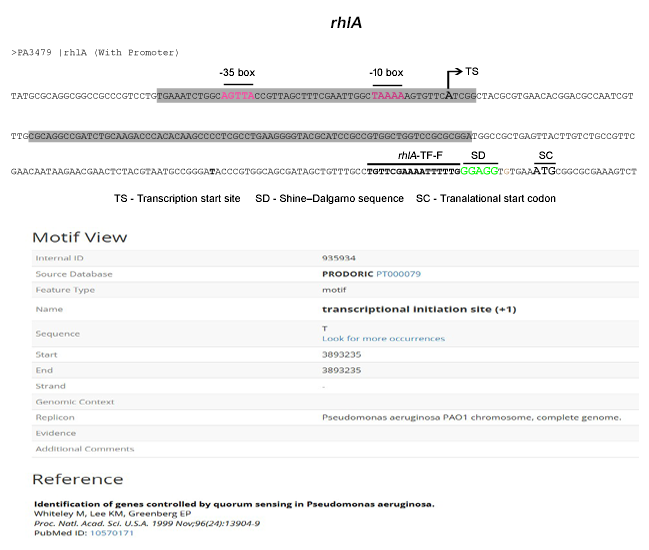
**

**Figure S5.** Primer positions in the 5'-UTR region relative to the transcription start site, Shine-Dalgarno sequence, and translational start codon of *rhlA* genes for the translational fusion study. The transcription start site was annotated based on the *Pseudomonas* genome database [6], with the reference source provided at the lower panel.

**Figure S6**

**
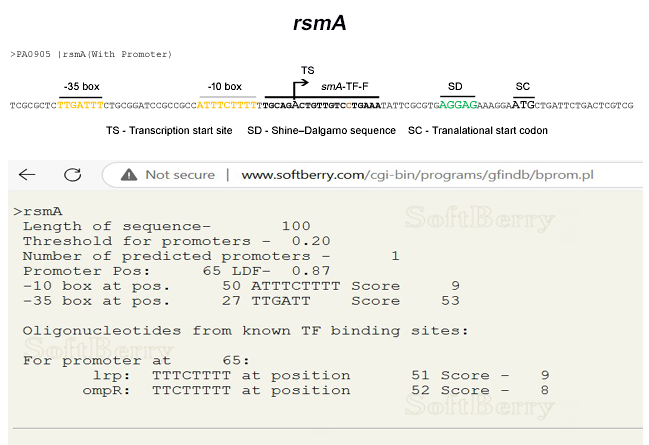
**

**Figure S6.** Primer positions in the 5'-UTR region relative to the transcription start site, Shine-Dalgarno sequence, and translational start codon of *rsmA* genes for the translational fusion study. The transcription start site was annotated using online promoter prediction tools, as no information was available in the *Pseudomonas* genome database [6]. The output results are provided at the lower panel.

**Figure S7**

**
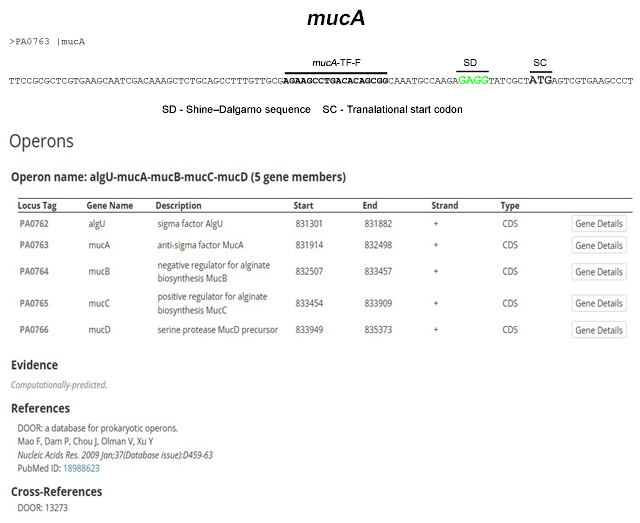
**

**Figure S7.** Primer positions in the 5'-UTR region relative to the Shine-Dalgarno sequence and translational start codon of *mucA* genes for the translational fusion study. The transcription start site was not annotated, because the *mucA* is at the second position in its operon, as described in the *Pseudomonas* genome database [6]. The specific reference source is provided at the lower panel.

**Figure S8**

**
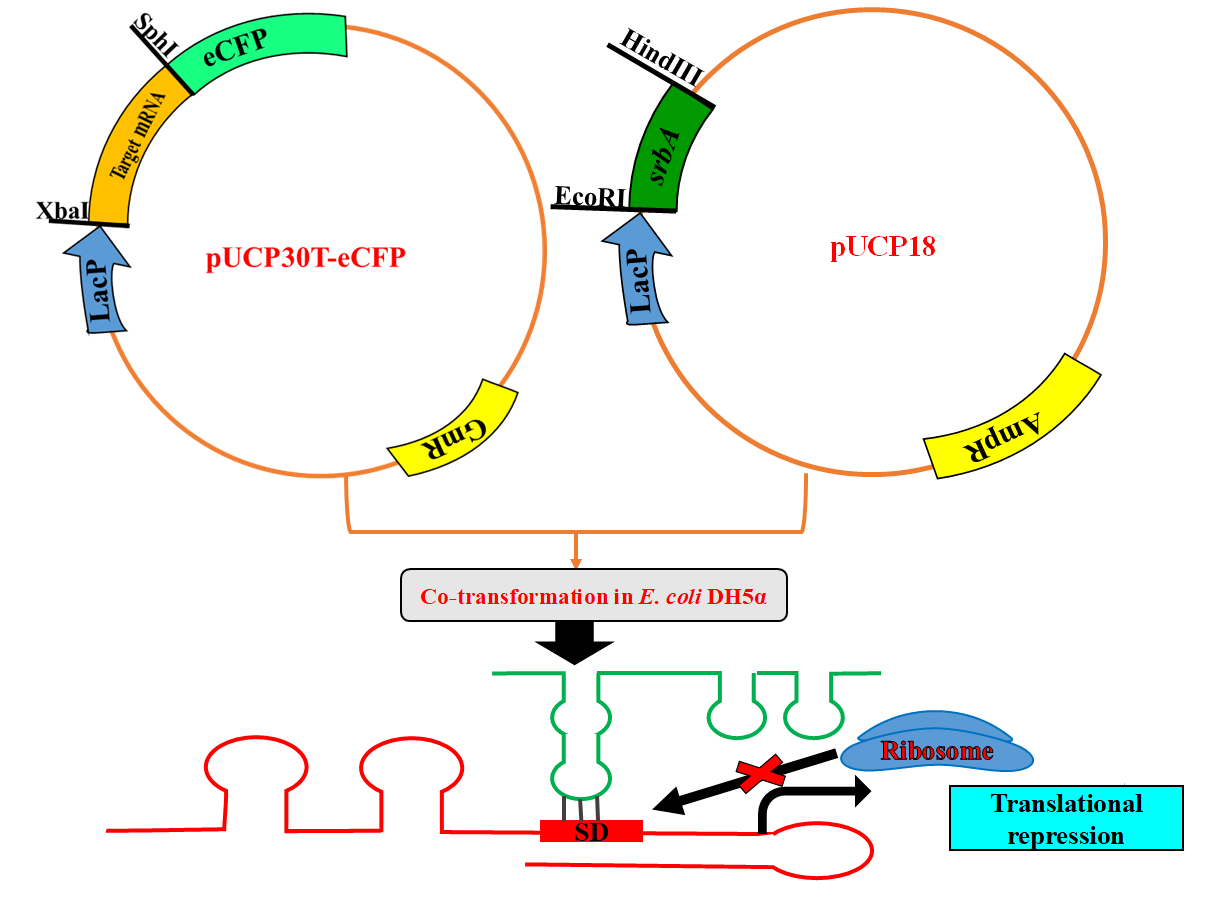
**

**Figure S8.** A schematic representation of translational repression was illustrated using a translation fusion assay with pUCP30T-*target gene*-eCFP, along with either pUCP18-SrbA^+^ or the empty vector pUCP18 (EV).

**Figure S9**

**
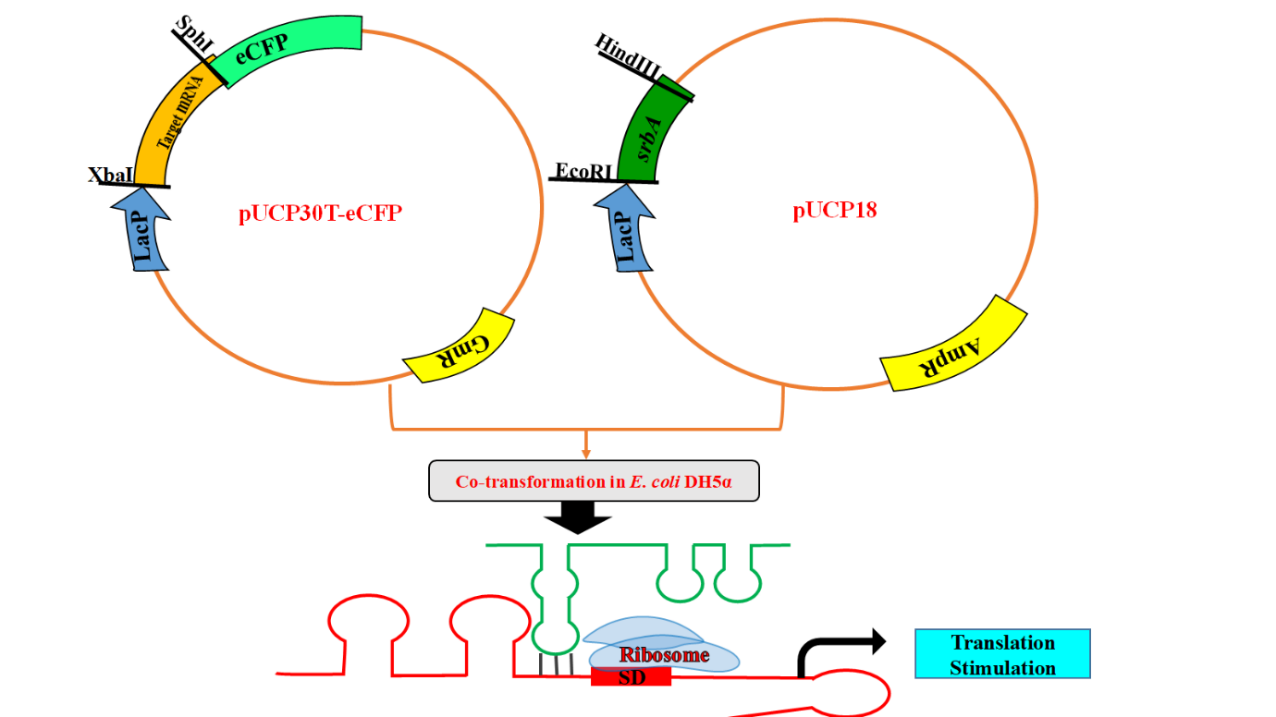
**

**Figure S9.** A schematic representation of translational stimulation was illustrated using a translation fusion assay with pUCP30T-*target gene*-eCFP, along with either pUCP18-SrbA^+^ or the empty vector pUCP18 (EV).

**Reference**

1. Saha, P., Mukherjee, S.K., Hossain, S.T., 2024. Regulation of TCA cycle genes by *srbA* sRNA: Impacts on *Pseudomonas aeruginosa* virulence and survival. *Biochem. Biophys. Res. Commun.* **737**, 150520. <https://doi.org/10.1016/j.bbrc.2024.150520>
2. Kar, A., Saha, P., De, R., Bhattacharya, S., Mukherjee S.K., Hossain, S.T. (2024) Unveiling the role of PA0730.1 sRNA in *Pseudomonas* *aeruginosa* virulence and biofilm formation: Exploring *rpoS* and *mucA* regulation. *Int. J. Biol. Macromol.* **279**, 135130. <https://doi.org/10.1016/j.ijbiomac.2024.135130>
3. Schweizer, H. P. (1991). *Escherichia*-*Pseudomonas* shuttle vectors derived from pUC18/19. *Gene* **97** (1), 109–121. <https://doi.org/10.1016/0378-1119(91)90016-5>
4. Chen, W., Zhang, Y., Zhang, Y., Pi, Y., Gu, T., Song, L., Wang, Y., Ji, Q. (2018). CRISPR/Cas9-based genome editing in *Pseudomonas* *aeruginosa* and cytidine deaminase-mediated base editing in *pseudomonas* species. *iScience* **6**, 222–231. <https://doi.org/10.1016/j.isci.2018.07.024>
5. Barbier, M., Damron, F.H. (2016) Rainbow vectors for broad-range bacterial fluorescence labelling. *PloS One* **11** (3), e0146827. [https://doi.org/10.1371/journal. pone.0146827](https://doi.org/10.1371/journal.%20pone.0146827)
6. Winsor, G.L., Griffiths, E.J., Lo, R., Dhillon, B.K., Shay, J.A. Brinkman, F.S. (2016) Enhanced annotations and features for comparing thousands of Pseudomonas genomes in the *Pseudomonas* genome database. Nucleic Acids Res. 44 (D1), D646–D653. <https://doi.org/10.1093/nar/gkv1227>
